# Causal Relationship Between Lung Function and Atrial Fibrillation: A Two Sample Univariable and Multivariable, Bidirectional Mendelian Randomization Study

**DOI:** 10.3389/fcvm.2021.769198

**Published:** 2021-11-11

**Authors:** Qiaoyun Zhang, Xiaoyu Zhang, Jie Zhang, Biyan Wang, Xiaoni Meng, Qiuyue Tian, Jinxia Zhang, Mengyang Jiang, Yiqiang Zhang, Deqiang Zheng, Lijuan Wu, Wei Wang, Baoguo Wang, Youxin Wang

**Affiliations:** ^1^Beijing Key Laboratory of Clinical Epidemiology, School of Public Health, Capital Medical University, Beijing, China; ^2^Department of Anesthesiology, Sanbo Brain Hospital, Capital Medical University, Beijing, China; ^3^Centre for Precision Health, Edith Cowan University, Joondalup, WA, Australia

**Keywords:** forced expiratory volume in one second (FEV1), forced vital capacity (FVC), the ratio of FEV1 over FVC (FEV1/FVC), atrial fibrillation (AF), bidirectional Mendelian randomization

## Abstract

**Background:** Observational studies have identified impaired lung function accessed by forced expiratory volume in one second (FEV1), forced vital capacity (FVC) or the ratio of FEV1 over FVC (FEV1/FVC) as an independent risk factor for atrial fibrillation (AF). However, the result may be affected by confounders or reverse causality.

**Methods:** We performed univariable MR (uvMR), multivariable MR (mvMR) and bidirectional two-sample MR to jointly estimate the causality of lung function with AF. Apart from the inverse variance weighted (IVW) approach as the main MR analysis, three complementary sensitive analyses approaches including MR-Egger regression, weighted median (WM) MR and Pleiotropy Residual Sum and Outlier (MR-PRESSO) in uvMR as well as mvMR-Egger and mvMR-PRESSO in mvMR were applied to control for pleiotropy. Linkage disequilibrium score (LDSC) regression was applied to estimate genetic correlation between lung function and AF.

**Results:** All forward and reverse uvMR analyses consistently suggested absent causal relations between lung function and AF risk [forward IVW: odds ratio (OR)_FEV1_ = 1.031, 95% CI = 0.909–1.169, *P* = 0.630; OR_FVC_ = 1.002, 95% CI = 0.834–1.204, *P* = 0.982; OR_FEV1/FVC_ = 1.076, 95% CI = 0.966–1.199, *P* = 0.182; reverse IVW: OR_FEV1_ = 0.986, 95% CI = 0.966–1.007, *P* = 0.187; OR_FVC_ = 0.985, 95% CI = 0.965–1.006, *P* = 0.158; OR_FEV1/FVC_ = 0.994, 95% CI = 0.973–1.015, *P* = 0.545]. The forward MR-Egger showed that each standard deviation (SD) increase in FEV1/FVC was related to a higher AF risk (OR = 1.502, 95% CI = 1.178–1.915, *P* = 0.006) without heterogeneity (Q_pval = 0.064), but pleiotropy effect exist (intercept = −0.017, *P* = 0.012). However, this significant effect disappeared after adjustment of FEV1 and FVC (OR = 1.523, 95% CI = 0.445–5.217, *P* = 0.503) in mvMR. No evidence was found for independent causal effects of FEV1 and FVC on AF in mvMR analysis by using mvIVW method (OR_FEV1_ = 0.501, 95% CI = 0.056–4.457, *P* = 0.496; OR_FVC_ = 1.969, 95% CI = 0.288–13.474, *P* = 0.490). Notably, the association between lung function and AF were replicated using the FinnGen cohort data.

**Conclusions:** Our findings reported no coheritability between lung function and AF, and failed to find substantial causal relation between decreased lung function and risk of AF. However, lung function and AF were both associated with inflammation, which may be potential pathway, warranting further study.

## Introduction

Atrial fibrillation (AF) is the most common persistent arrhythmia and its prevalence increases with age (1). With the aging of global population, AF with increased morbidity and mortality has become an increasing public health burden (1–3). Published observational studies (4–9) based on different ethnic-groups indicated that declined lung function is related to higher AF incidence.

Lung function accessed by forced expiratory volume in one second (FEV1), forced vital capacity (FVC) and the ratio of FEV1 over FVC (FEV1/FVC) are used to predict morbidity and mortality associated with different airway disease (10–12) and all-cause mortality (13). Compared with individuals in sinus rhythm, more patients with persistent AF had lung function below normal range (14). Meanwhile, higher AF prevalence was found in subjects with severe impairment of lung function than in those without or with mild/moderate impairment of lung function (5).

The prevalence of AF and impaired lung function is increasing worldwide and seriously affect the quality of life (15, 16), so clarifying the direction and underlying causality of these associations would be helpful to guide prevention. Age, gender, height and smoking are strongly correlated with lung function and AF, which are particularly important confounders (17–19). Furthermore, due to the inter-correlations among different lung function traits, identifying the causal effect of lung function on AF is challenging and vice versa.

Mendelian randomization (MR) study may provide more credible evidence concerning the causality of lung function and AF. Genetic variants robustly related to lung function or AF would be used as instrumental variables (IVs), respectively. Due to the random allocation from parents to their offspring at conception, IVs are less susceptible to confounders or reverse causality (20, 21). Multivariable MR (mvMR) integrates a series of pleiotropic SNPs (22) associated with at least one risk factors as IVs to evaluate the causal influence of each risk factor on the outcome. In addition, causal relation can be estimated even if no variation shows a specific association with any individual risk factor in the case of horizontal pleiotropy (23). Linkage disequilibrium score (LDSC) regression could further explore the association between lung function and AF by evaluating the genetic correlation (24). A recent MR study (25) investigated the causal inference of FEV1 and FVC on cardiovascular diseases (CVDs) including AF. However, that study did not consider the role of FEV1/FVC, the inter-correlation of different lung function traits as well as the independency of dataset of the SNP-AF and SNP-lung function. Therefore, we integrated the uvMR, mvMR and bidirectional MR approaches to identify causal relation between lung function and AF using summary statistics GWAS results in European population. Our study complements published MR studies by including more lung function traits and considering the overlap of datasets.

## Methods

### Data Sources

Since this study only used publicly available summary statistic from relevant genome-wide-association studies (GWAS) and did not use the individual data, ethical approval was not required.

#### GWAS of AF

The study of Biobank-based GWAS tested association between more than 30 million genetic variants and AF in 1,030,836 individuals (60,620 cases and 970,216 controls) of European ancestry from the Nord-Trøndelag Health Study (HUNT), deCODE, the Michigan Genomics Initiative (MGI), DiscovEHR, UK Biobank, and the AFGen Consortium. Full details are provided elsewhere (26).

#### GWAS of Lung Function (FEV1, FVC, and FEV1/FVC)

Summary genetic association estimates for lung function were obtained from the largest currently available GWAS study in 400,102 individuals (UK Biobank: 321,407, SpiroMeta: 79,055) of European ancestry and were adjusted for age, age^2^, gender, height, and smoking status. Given the datasets for AF included individuals from UK Biobank, we just selected SpiroMeta results from lung function GWAS to perform MR analyses. Because the independence and non-overlapping of exposure and outcome is a prominent prerequisite for the validity of the two sample MR method (27). Full details are provided elsewhere (28) and summary data are available online *via* LD-Hub (http://ldsc.broadinstitute.org/ldhub/).

Considering the stability of our results, we replicated the two-sample MR analysis using the above lung function GWAS data including UK Biobank participants (SpiroMeta: 79,055 and UK Biobank: 321,407) and another latest publicly available data of AF (22,068 cases and 196,724 controls) released from the FinnGen cohort (Release 5, https://www.finngen.fi/en).

### Instrumental Variables for Lung Function and AF

To interpret the resulting estimates as causal effects, three common assumptions of IV estimation must hold: (1) the IV is associated with the exposure robustly, (2) the IV is independent of confounders, and (3) the IV influences the outcome exclusively *via* the exposure (27).

We performed MR using genetic variants (significance of SNPs: *P* < 5 × 10^−8^) associated with AF as IVs to investigate their effect on lung function (FEV1, FVC, and FEV1/FVC), and vice versa. Among others, violations of the second and third assumption occur in case of population stratification, when different ethnic subgroups are present with different allele frequencies. We overcame this issue by focusing on individuals with European ancestry (29). Violations of the third assumption occur in case of linkage disequilibrium (LD) between the included SNPs or in case of pleiotropy. Horizontal pleiotropy, i.e., the same SNP independently influences multiple phenotypes, leads to violations of the third assumption and to biased estimates (27). In view of that, only independent variants (LD, *r*^2^ = 0.0001) based on European ancestry reference data from the 1,000 Genomes Project were retained.

In brief, the process for selecting IVs was as follows: we first extract significant SNPs (*P* < 5 × 10^−8^) from exposure GWAS; then prune SNPs for linkage disequilibrium (*r*^2^ = 0.0001); next, extract the retained SNPs from the outcome datasets; last, harmonize the exposure and outcome SNPs (e.g., removing the SNPs for being palindromic with intermediate allele frequencies).

The mvMR approach takes into account the inter-relationship between FEV1, FVC, and FEV1/FVC and the IVs used in the mvMR analyses are often associated with all exposures. This method is based on the above-mentioned standard IVs method which allow for multiple exposures. We selected independent SNPs (*r*^2^ < 0.0001) that were associated with FEV1, FVC, or FEV1/FVC at *P* < 5 × 10^−8^; then we merged all SNPs related to any lung-function traits by removing duplicate SNPs with higher *p*-values; next, we extracted the relevant information of the merged SNPs from the original exposure datasets; finally, we used the last extracted SNPs as IVs for mvMR analysis.

We estimated the *R*^2^ [*R*^2^ = 2×EAF× (1-EAF) × Beta^2^] of each SNP and summed them up to calculate the overall *R*^2^ and *F* statistics [*R*^2^ × (N-2)/(1-R^2^); N represents the number of individuals in the exposure GWAS] using the sample size of exposure GWAS (30). Higher *R*^2^ and *F* statistic indicate lower risk of weak IVs bias (31).

### LDSC Regression Analysis

LDSC regression regresses χ^2^ statistics for two phenotypes to estimate SNP based coheritability on LDSCs. Genetic correlations between lung function and AF can be evaluated by the regression slope (24).

### MR Analyses

For uvMR, we used inverse variance weighted (IVW) method as the main analysis. The IVW analysis does not consider the intercept (constrained to zero) in the regression and uses the reciprocal of the outcome variance as the weight for fitting (32). Results may be biased if IVs exhibit horizontal pleiotropy, influencing outcomes by other pathways other than the exposure (33). Therefore, we supplemented sensitivity analyses, including the MR-Egger regression and weighted median (WM). The MR-Egger regression analysis allows free evaluation of the intercept (not set to zero) as an indicator of average pleiotropic bias (33) and gives a consistent estimate when all genetic variants are invalid IVs. If more than half of the weight derived from effective IVs, the WM method may come to an unbiased estimate of causality (34). For mvMR, we used the extension of the IVW approach (variants uncorrected, random effect model) which performed multivariable weighted linear regression (33), and the extension of the MR-Egger regression approach to correct for both measured and unmeasured pleiotropy in mvMR (35).

To evaluate the robustness of significant results, we conducted leave-one-out sensitivity analysis, the Cochran *Q* heterogeneity test and *I*^2^ statistics (36), and the MR Egger intercept test (37) on horizontal pleiotropy to detect heterogeneity. In addition, horizontal pleiotropy can be evaded by applying mvMR approaches (i.e., by considering the overlapping IVs directly in the estimation) (23). Pleiotropy Residual Sum and Outlier (MR-PRESSO) was applied to detect and correct for outlier SNPs reflecting pleiotropic biases (38).

The MR results are expressed as odds ratios (ORs) with 95% confidence intervals (CIs) interpreted as AF risk per SD increase in log odds of the lung function traits (or alternatively, the risk of impaired lung function per SD increase in log odds of AF). We use a two-sided α of 0.017 based on testing three lung function traits against AF outcome to define significant or not. MR analyses were performed using the packages “MendelianRandomization,” “TwoSampleMR,” and “MR-PRESSO” packages (https://cran.r-project.org/package) in the Rstudio (R version 4.0.3, R Project for Statistical Computing). Power calculation was performed using mRnd developed by Brion et al. (39) (https://cnsgenomics.com/shiny/mRnd/).

## Results

### LDSC Regression

We performed LDSC analysis to estimate the genetic correlation between lung function and AF as well as different lung-function traits using LDSC software: https://github.com/bulik/ldsc. There was no evidence for genetic correlations of AF with all lung function traits (rg = NA, *P* = NA), namely no coheritability between two traits. There was evidence for higher genetic correlations of FEV1 with FVC and FEV1/FVC (FEV1 vs. FVC: rg = 0.8717, *P* = 0.0; FEV1 vs. FEV1/FVC: rg = 0.3859, *P* = 2.93e-14; FVC vs. FVC: rg = −0.1287, *P* = 0.0144) (Table 1).

**Table 1:** Genetic Correlation estimates from LDSC regression.

**Phenotype 1**	**Phenotype 2**	**Rg**	**se**	***P***
FEV1	AF	NA	NA	NA
FVC	AF	NA	NA	NA
FEV1/FVC	AF	NA	NA	NA
FEV1	FEV1/FVC	0.3859	0.0508	2.93e−14
FEV1	FVC	0.8717	0.0167	0.0
FVC	FEV1/FVC	−0.1287	0.0526	0.0144

*FEV1, forced expiratory volume in one second; FVC, forced vital capacity; FEV1/FVC, the ratio of FEV1 over FVC; AF, atrial fibrillation*.

### Genetic IVs Selection and Validation (Original Analyses)

In the univariable bidirectional MR analyses, we obtained 18/11/10/93 LD-independent SNPs which achieved genome wide significance (*P* < 5 × 10^−8^) for FEV1/FVC, FEV1, FVC, and AF, respectively, then we extracted those SNPs from corresponding outcome dataset. Finally, 16/9/8/82 SNPs remained after harmonizing the datasets of exposure and outcome (2/2/2/11 palindrome SNPs with intermediate allele frequencies were removed from FEV1/FVC, FEV1, FVC, and AF).

**Figure 1:**
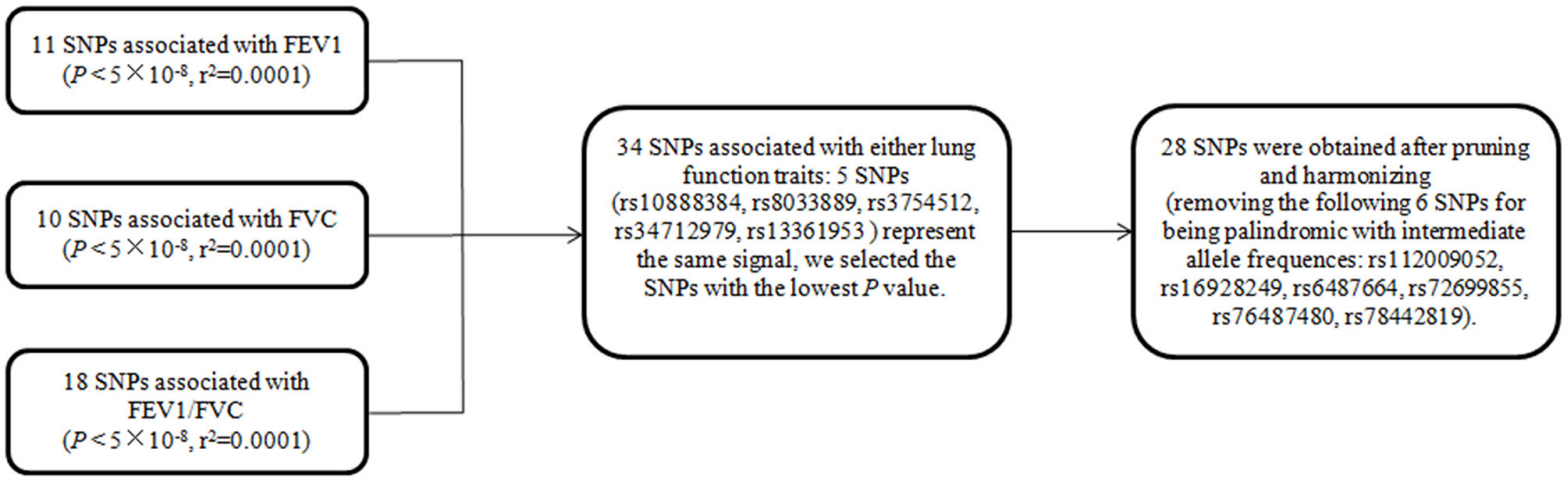
The flow chart of SNP selection for multivariate MR. SNPs are a set of instrumental variable related to FEV1 and/or FVC and/or FEV1/FVC. FEV1, forced expiratory volume in one second; FVC, forced vital capacity; FEV1/FVC, the ratio of FEV1 over FVC; SNP, single nucleotide polymorphism.

In mvMR analysis, all 39 (11+10+18) above-mentioned LD-independent (*r*^2^ < 0.0001) SNPs associated with FEV1, FVC, and FEV1/FVC at *P* < 5 × 10^−8^ were included. Five of these SNPs (rs10888384, rs8033889, rs3754512, rs34712979, rs13361953) represent the same signal, so we aggregate a list of SNP (34 = 39 – 5) by selecting the SNPs with the lowest *P*-value. This resulted in 34 SNPs, of which seven were related to FEV1 but not FVC and FEV1/FVC at *P* < 5 × 10^−8^, 14 were associated with FEV1/FVC but not FEV1 and FVC at *P* < 5 × 10^−8^, eight were associated with FVC but not FEV1 and FEV1/FVC at *P* < 5 × 10^−8^, and five were associated with any two traits at *P* < 5 × 10^−8^. Then, we extracted relevant information of 34 SNPs from the original exposure datasets, respectively. Finally, 28 (28 = 34–6, six palindrome SNPs with intermediate allele frequencies were removed) SNPs for any lung function trait were obtained after harmonizing with SNP-outcome (the flow chart of SNPs selection was shown in Figure 1).

The number of SNPs for FEV1 [nine, variance explained (*R*^2^): 0.6%], FVC [eight, variance explained (*R*^2^): 0.5%], FEV1/FVC [16, variance explained (*R*^2^): 1.3%] were small and the statistical power was insufficient. However, the *F* statistics (>10, ranging from 32.7 to 21,355.5) for each SNP and the power of IVs in the mvMR (28, variance explained (*R*^2^): 2%; power: 100%) were sufficient, indicating that those SNPs satisfy the strong relevance assumption and the weak IV bias would not likely affect the causal inference. The number of IVs included for exposures were listed in Table 2, and details for the characteristics of SNPs used in uvMR and mvMR were shown in Supplementary Tables 1A–C, respectively.

**Table 2:** Baseline information of exposures used in our analyses.

**Exposure**		**Outcome**		**Number of IVs**		***R*** ^**2**^		**Power (%)**		**F_statistics**	
**Trait**	**Sample**	**Trait**	**Sample**	**mvMR**	**uvMR**	**mvMR**	**uvMR**	**mvMR**	**uvMR**	**mvMR**	**uvMR**
FEV1	79,055	AF	1,030,836		9		0.0059		4		467.76
FVC	79,055	AF	1,030,836	28	8	0.0198	0.0045	100	2	1596.87	355.65
FEV1/FVC	79,055	AF	1,030,836		16		0.0125		38		991.84
AF	1,030,836	FEV1	79,055	–	82	–	0.1866	–	100	–	163,697.38
AF	1,030,836	FVC	79,055	–	82	–	0.1866	–	100	–	163,697.38
AF	1,030,836	FEV1/FVC	79,055	–	82	–	0.1866	–	100	–	163,697.38

*FEV1, forced expiratory volume in one second; FVC, forced vital capacity; FEV1/FVC, the ratio of FEV1 over FVC; AF, atrial fibrillation; IVs, instrumental variables; R^2^, Variance explained by the SNPs on exposure*.

### Genetic IVs Selection and Validation (Replication Analyses)

According to the same method as above, we obtained 167/161/186/25 SNPs for FEV1, FVC, FEV1/FVC, and AF, respectively, in the uvMR replication analyses, and 470 SNPs for lung function in the mvMR analyses. They were described in detail in the Supplementary Material.

### Causal Association of FEV1, FVC, or FEV1/FVC With AF *via* Forward MR (Original Analyses)

#### FEV1

**Figure 2:**
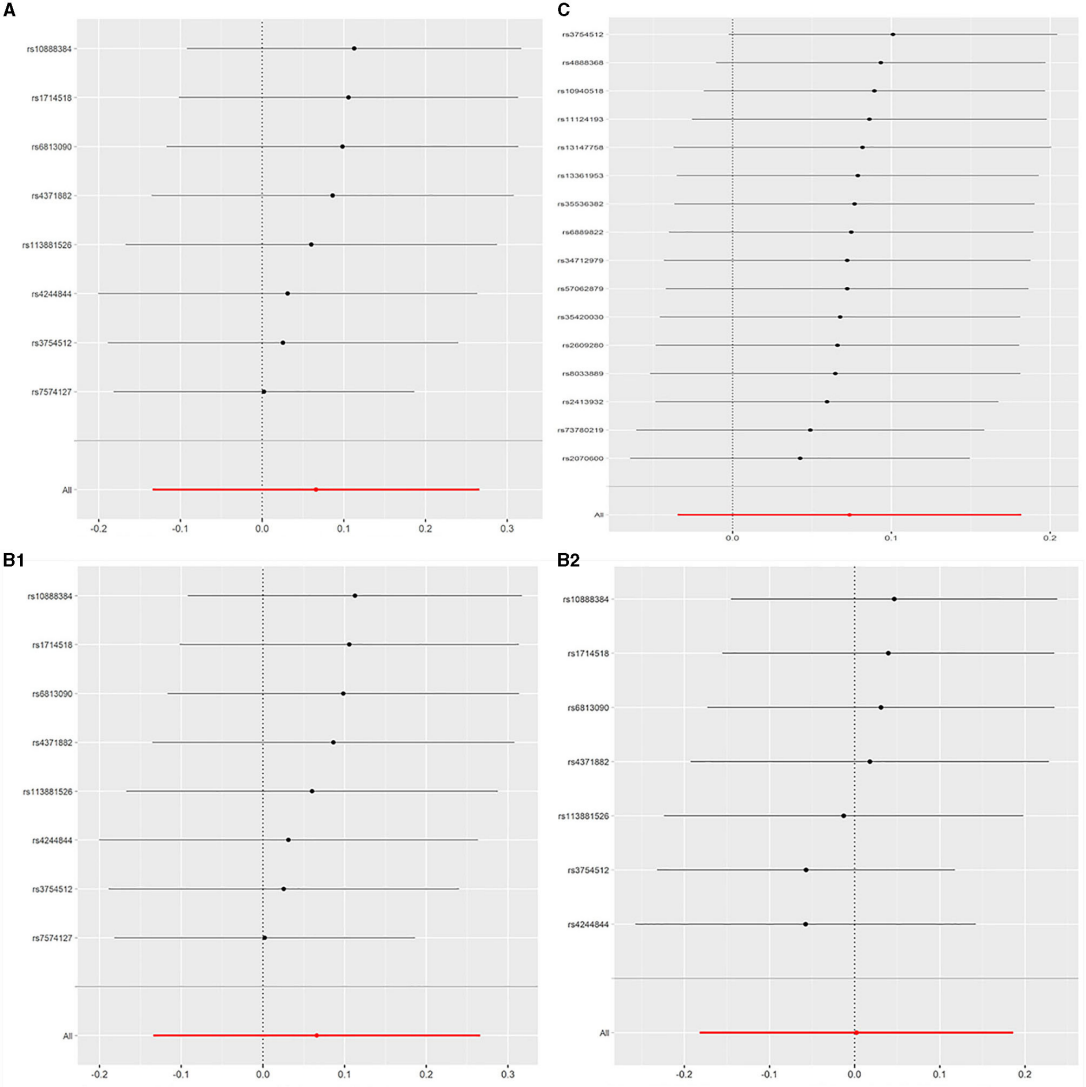
**(A)** Leave-one-out plot: MR sensitivity analysis for FEV1 on AF. **(B1)** Leave-one-out plot: MR sensitivity analysis for FVC on AF (not removing the outlier SNP). **(B2)** Leave-one-out plot: MR sensitivity analysis for FVC on AF (removing the outlier SNP). **(C)** Leave-one-out plot: MR sensitivity analysis for FEV1/FVC on AF. Each row is interpreted as a Mendelian randomization analysis of the causal effects of FEV1 **(A)**, FVC **(B)**, and FEV1/FVC **(C)** on AF using the remaining instrumental variables other than the SNP listed on the y-axis. The dots represent the odds ratio after removing the corresponding SNP, and the lines represent corresponding 95% confidence interval. FEV1, forced expiratory volume in one second; FVC, forced vital capacity; FEV1/FVC, the ratio of FEV1 over FVC; AF, atrial fibrillation; SNP, single nucleotide polymorphism.

**Figure 3:**
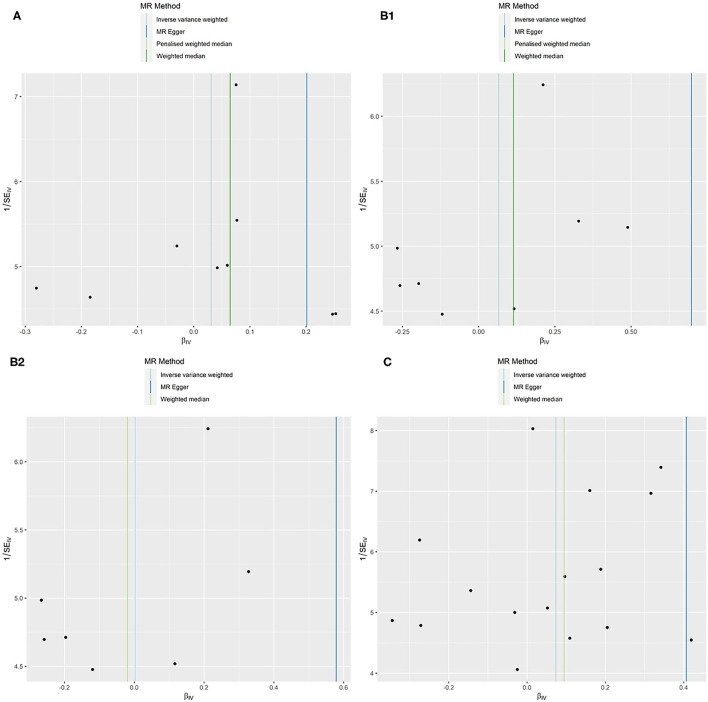
MR funnel plots: FEV1 for AF **(A)**; FVC for AF **(B1)**: not removing the outlier SNP; **(B2)**: removing the outlier SNP; FEV1/FVC for AF **(C)**.

All models suggested that FEV1 has no causal effect on AF (OR = 1.031, 95% CI = 0.909–1.169, *P* = 0.630) without evidence of heterogeneity (Q_pval = 0.719, *I*^2^ = 0%) nor pleiotropy effect (intercept = −0.006, *P* = 0.565). The WM method estimates were more precise than the MR-Egger method (WM: OR = 1.067, 95% CI = 0.905–1.258, *P* = 0.442; MR Egger: OR = 1.223, 95% CI = 0.694–2.153, *P* = 0.509). The leave-one-out sensitivity analysis (Figure 2A) indicated that no SNP altered the MR estimates, suggesting the stability and reliability of the forward MR results. The MR-PRESSO test detected no SNP as pleiotropic outlier (Table 3 and Supplementary Tables 2, 3). The funnel plot indicated no evidence of asymmetry, so there was no directional pleiotropy (Figure 3A).

**Table 3:** Forward causal relations of FEV1, FVC, and FEV1/FVC with AF performed by uvMR.

**Exposure**	**nSNPs**	**OR (95%CI)**	***P***	**Q_pval (*I^**2**^*)**	**Intercept (*P*)**	**Global *P***
FEV1 vs. AF						
IVW	9	1.031 (0.909–1.169)	0.630	0.719(0)		
MR-Egger	9	1.223 (0.694–2.153)	0.509		−0.006 (0.565)	
WM	9	1.067 (0.905–1.258)	0.442			
MR-PRESSO	9		0.776			0.242
FVC vs. AF						
IVW	7	1.002 (0.834–1.204)	0.982	0.158 (0.35)		
MR-Egger	7	0.783 (0.604–5.266)	0.343		−0.022 (0.339)	
WM	7	0.981 (0.807–1.192)	0.844			
MR-PRESSO	7		0.421			0.080
FEV1/FVC vs. AF						
IVW	16	1.076 (0.966–1.199)	0.182	0.064(0.38)		
MR-Egger	16	1.052 (1.178–1.195)	0.006		−0.017 (0.012)	
WM	16	1.099 (0.969–1.246)	0.139			
MR-PRESSO	16		0.986			0.007

*nSNPs, number of single nucleotide polymorphisms; OR, odds ratio; CI, confidence interval; Q_pval, P-value of the Cochran Q statistic; I^2^ = (Q-df)/Q × 100%; P < 0.017 were considered statistically significant. FEV1, forced expiratory volume in one second; FVC, forced vital capacity; FEV1/FVC, the ratio of FEV1 over FVC; AF: atrial fibrillation. uvMR, univariable Mendelian randomization; IVW, inverse-variance weighted; WM, weighted median; MR-PRESSO, Pleiotropy Residual Sum and Outlier*.

#### FVC

No evidence was detected for a causal relation between FVC and AF in IVW method (OR = 1.068, 95% CI = 0.874–1.305, *P* = 0.519) with no evidence of pleiotropy (intercept = −0.024, *P* = 0.361) but moderate heterogeneity (Q_pval = 0.039, *I*^2^ = 52%). The leave-one-out sensitivity analysis (Figure 2B1) and the MR-PRESSO test both detected the third SNP (rs7574127) as pleiotropic outlier. Although significant heterogeneity was no longer exist (Q_pval = 0.158, *I*^2^ = 35%) after rerun the MR by removing the outlier SNP, the MR estimates did not alter significantly (IVW: OR = 1.002, 95% CI = 0.834–1.204, *P* = 0.982; intercept = −0.022, *P* = 0.339) (Table 3 and Supplementary Tables 2, 3 and Figure 2B2). No significant asymmetry was found in the funnel chart, either before or after the outlier SNP removed (Figures 3B1,B2).

#### FEV1/FVC

All methods except MR Egger regression suggested that FEV1/FVC has no causal effect on AF (IVW: OR = 1.076, 95% CI = 0.966–1.199, *P* = 0.182; WM: OR = 1.099, 95% CI = 0.969–1.246, *P* = 0.139) without evidence of heterogeneity (Q_pval = 0.064, *I*^2^ = 38%). Although MR Egger estimate was significant (OR = 1.502, 95% CI = 1.178–1.915, *P* = 0.006), overall horizontal pleiotropic effects existed (intercept = −0.017, *P* = 0.012), highlighting the need for mvMR analysis. The leave-one-out sensitivity analysis (Figure 2C) and the MR-PRESSO test both showed that no significant outliers derived the majority of the association signal (Table 3 and Supplementary Tables 2, 3). The funnel plot indicated no evidence of asymmetry (Figure 3C).

### Causal Association of AF With FEV1, FVC, and FEV1/FVC *via* Reverse MR (Original Analyses)

**Figure 4:**
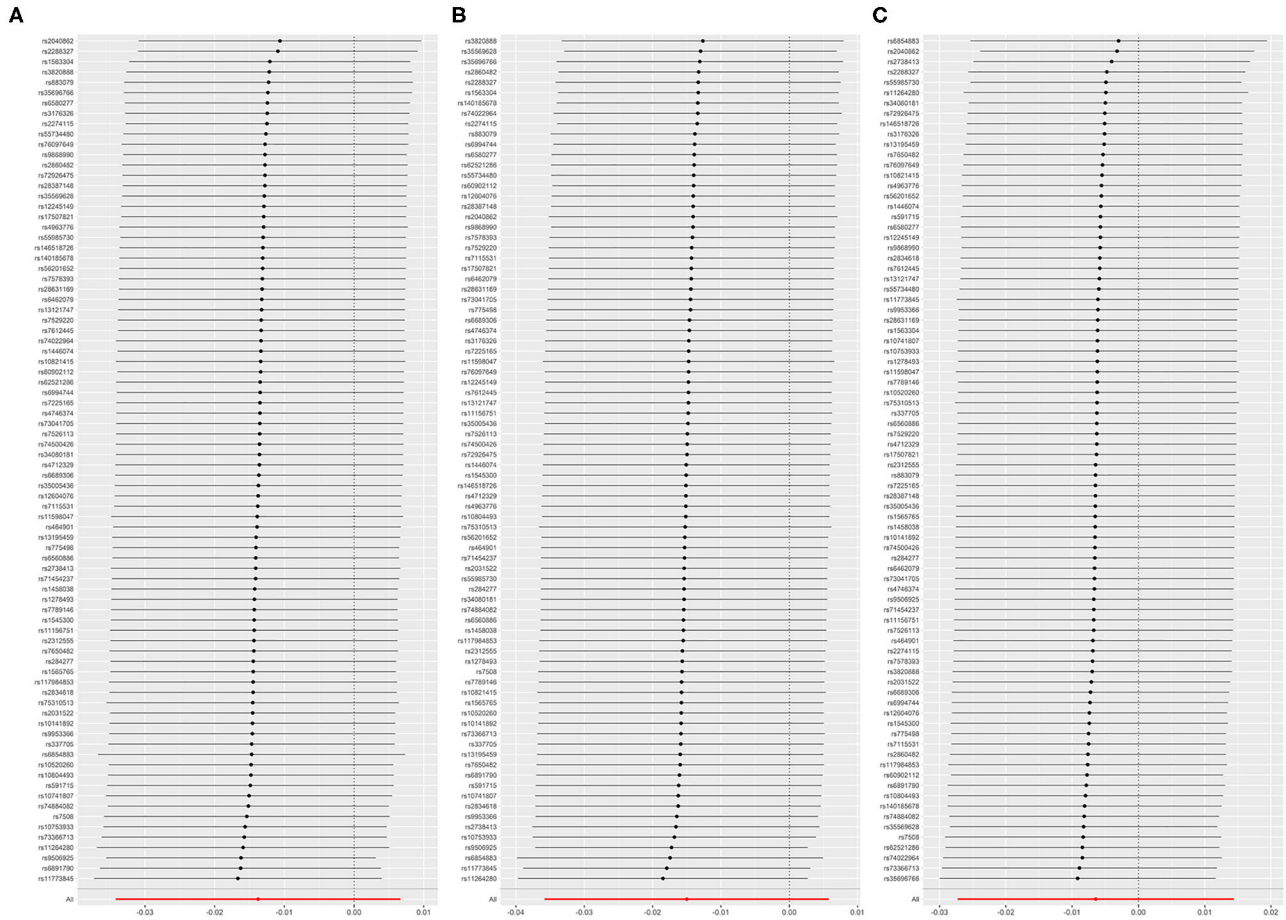
**(A)** Leave-one-out plot: MR sensitivity analysis for AF on FEV1. **(B)** Leave-one-out plot: MR sensitivity analysis for AF on FVC. **(C)** Leave-one-out plot: MR sensitivity analysis for AF on FEV1/FVC. Each row is interpreted as a Mendelian randomization analysis of the causal effects of AF on FEV1 **(A)**, FVC **(B)**, and FEV1/FVC **(C)** using the remaining instrumental variables other than the SNP listed on the y-axis. The dots represent the odds ratio after removing the corresponding SNP, and the lines represent the corresponding 95% confidence interval. FEV1, forced expiratory volume in one second; FVC, forced vital capacity; FEV1/FVC, the ratio of FEV1 over FVC; AF, atrial fibrillation; SNP, single nucleotide polymorphism.

**Figure 5:**
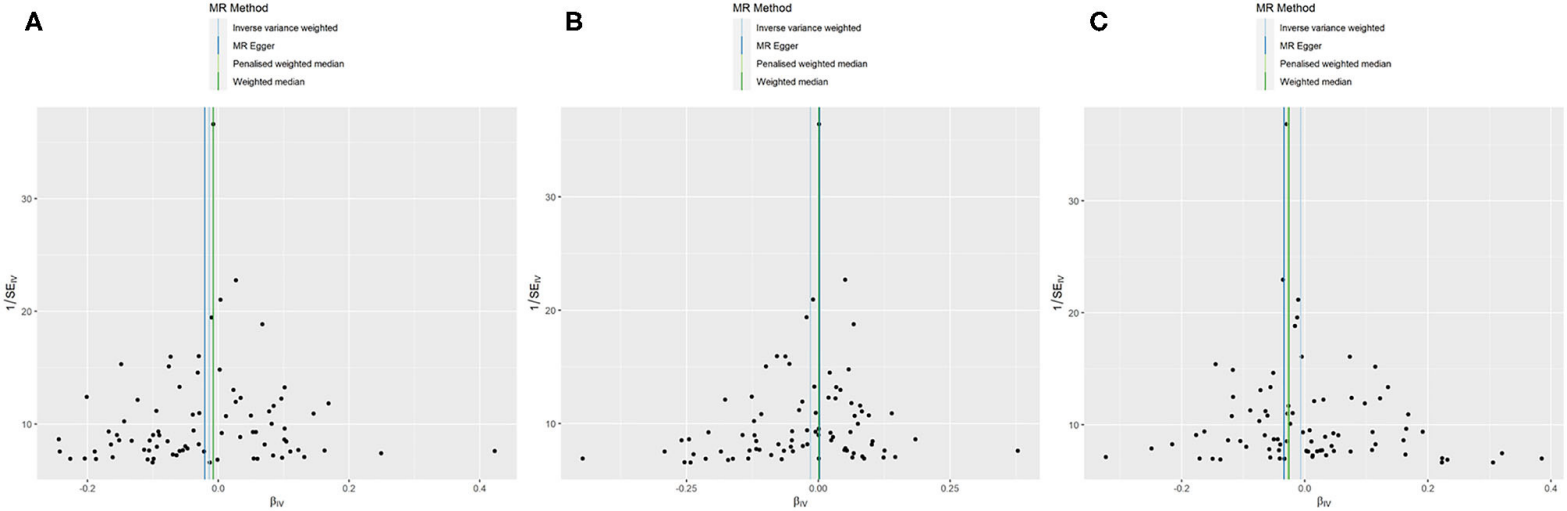
MR funnel plots: AF for FEV1 **(A)**; AF for FVC **(B)**; AF for FEV1/FVC **(C)**.

All models in reverse MR analyses consistently suggested that genetically instrumented AF has no significant correlation with FEV1 (OR = 0.986, 95% CI = 0.966–1.007, *P* = 0.187), FVC (OR = 0.985, 95% CI = 0.965–1.006, *P* = 0.158) and FEV1/FVC (OR = 0.994, 95% CI = 0.973–1.015, *P* = 0.545). Pleiotropy bias (intercept_FEV1/FVC_ = 0.002, *P*_FEV1/FVC_ = 0.199; intercept_FEV1_ = 0.001, *P*_FEV1_ = 0.747; intercept_FVC_ = −0.001, *P*_FVC_ = 0.419) was not detected and heterogeneity (FEV1: Q_pval = 0.213, *I*^2^ = 11%; FVC: Q_pval = 0.162, *I*^2^ = 13%; FEV1/FVC: Q_pval = 0.127, *I*^2^ = 15%) was not found. These main estimates from IVW were broadly consistent with estimates from the WM and MR-Egger sensitivity analyses. All reverse leave-one-out sensitivity analyses (Figures 4A–C) and the MR-PRESSO tests indicated that the correlation between AF with FEV1, FVC, or FEV1/FVC was not significantly affected by any individual SNP, indicating the robustness of the reverse MR analyses results (Table 4 and Supplementary Tables 4, 5). The funnel plot indicated no evidence of asymmetry, so there was no directional pleiotropy (Figures 5A–C).

**Table 4:** Reverse causal relations of AF with FEV1, FVC, and FEV1/FVC performed by uvMR.

**Exposure**	**nSNPs**	**OR (95%CI)**	***P***	**Q_pval (*I^**2**^*)**	**Intercept (*P*)**	**Global *P***
AF vs. FEV1						
IVW	82	0.986 (0.966–1.007)	0.187	0.213 (0.11)		
MR-Egger	82	0.979 (0.936–1.026)	0.384		0.001 (0.747)	
WM	82	0.992 (0.959–1.026)	0.653			
MR-PRESSO	82		0.690			0.076
AF vs. FVC						
IVW	82	0.985 (0.965–1.006)	0.158	0.162 (0.13)		
MR-Egger	82	1.002 (0.957–1.050)	0.922		−0.001 (0.419)	
WM	82	1.001 (0.969–1.034)	0.954			
MR-PRESSO	82		0.239			0.084
AF vs. FEV1/FVC						
IVW	82	0.994 (0.973–1.015)	0.545	0.127 (0.15)		
MR-Egger	82	0.967 (0.923–1.013)	0.157		0.002 (0.199)	
WM	82	0.975 (0.943–1.007)	0.123			
MR-PRESSO	82		0.484			0.190

*nSNPs, number of single-nucleotide polymorphisms; OR, odds ratio; CI, confidence interval; Q_pval, P-value of the Cochran Q statistic; I^2^ = (Q-df)/Q × 100%; P < 0.017 were considered statistically significant. FEV1, forced expiratory volume in one second; FVC, forced vital capacity; FEV1/FVC, the ratio of FEV1 over FVC; AF, atrial fibrillation. uvMR, univariable Mendelian randomization; IVW, inverse-variance weighted; WM, weighted median; MR-PRESSO, Pleiotropy Residual Sum and Outlier*.

### Causal Association of Lung Function With AF *via* mvMR Approach (Original Analyses)

The estimate effects from the mvMR analysis showed that the direct effect of FEV1 or FVC (OR_FEV1_ = 0.501, 95% CI = 0.056–4.457, *P* = 0.496; OR_FVC_ = 1.969, 95% CI = 0.288–13.474, *P* = 0.490) controlling for another two lung function arguments was similar with the univariable setting. Additionally, no effect of FEV1/FVC (OR = 1.523, 95% CI = 0.445–5.217, *P* = 0.503) on AF could be observed, which was inconsistent with the univariable MR Egger result. Consistent with standard IVW regression results, mvMR-Egger regression (orientated to FEV1/FVC) and MR-PRESSO (detect no significant outliers) results also showed no significant association between lung function and AF, suggesting that the mvMR method successfully overcomes the bias caused by horizontal pleiotropy in the univariable MR analysis (Table 5 and Supplementary Table 6).

**Table 5:** Multivariable MR analyses for lung function and AF.

**Exposure**	**nSNPs**	**OR (95% CI)**	***P***	**Intercept *P***	**Q_pval (*I^**2**^*)**	**PRESSO *P***
FEV1						
mvIVW	28	0.501 (0.056–4.457)	0.496			0.502
mvMR-Egger (orientated to FEV1)	28	1.478 (0.201–10.873)		0.886		
FVC						
mvIVW	28	1.969 (0.288–13.474)	0.490		0.023 (0.634)	0.496
mvMR-Egger (orientated to FEV1)	28	1.247 (0.335–4.512)		0.361		
FEV1/FVC						
mvIVW	28	1.523 (0.445–5.217)	0.503			0.509
mvMR-Egger (orientated to FEV1)	28	0.800 (0.091–7.033)		0.299		

*FEV1, forced expiratory volume in one second; FVC, forced vital capacity; FEV1/FVC, the ratio of FEV1 over FVC; AF, atrial fibrillation; SE, standard error. mvIVW, multivariable inverse-variance weighted; mvMR, multivariable Mendelian randomization; MR-PRESSO, Pleiotropy Residual Sum and Outlier; OR, odds ratio; CI, confidence interval; nSNPs, number of single nucleotide polymorphisms*.

### Results of the Replication MR Analyses

In the replication MR analyses, there were more IVs and greater statistical power for lung function but less IVs for AF, and the MR results were basically consistent with original results (Table 6). We presented the detailed results as Supplementary Material.

**Table 6:** Main results of the replication MR analyses.

**Exposure**	**nSNPs**	**OR (95%CI)**	**P**	**Q_pval (*I^**2**^*)**
FEV1 vs. AF	159	0.844 (0.735–0.969)	0.016	0.035 (0.18)
FVC vs. AF	155	0.906 (0.781–1.052)	0.197	0.029 (0.18)
FEV1/FVC vs. AF	185	1.021 (0.930–1.122)	0.657	0.469 (0.00)
AF vs. FEV1	24	0.991 (0.980–1.002)	0.124	0.014 (0.43)
AF vs. FVC	19	0.947 (0.878–1.022)	0.161	0.602 (0.00)
AF vs. FEV1/FVC	12	0.997 (0.896–1.110)	0.956	0.282 (0.17)
FEV1				
FVC vs. AF	470	0.375 (0.038–3.693)	0.400	0.473 (0.002)
FEV1/FVC				

*nSNPs, number of single nucleotide polymorphisms; OR, odds ratio; CI, confidence interval; Q_pval, P-value of the Cochran Q statistic; I^2^ = (Q-df)/Q × 100%; P < 0.017 were considered statistically significant. FEV1, forced expiratory volume in one second; FVC, forced vital capacity; FEV1/FVC, the ratio of FEV1 over FVC; AF, atrial fibrillation*.

## Discussion

By performing univariate, multivariate and bidirectional MR analyses jointly using non-overlapping summary statistics for lung function and AF, as well as replicating MR analyses using lung function data including UK Biobank participants and FinnGen cohort data, we found that the correlation between impaired lung function and the risk of AF in published observational studies was not supported as a causal relationship. These results were consistent with a series of sensitivity analyses that were used to explore possible bias caused by horizontal pleiotropy.

The anatomical and physiological continuity of the lung, heart and vessels intuitively indicates that damage to any component of lung function may affect cardiovascular health (40), and vice versa. The findings of most observational studies indicate an inverse relation of lung function with AF. However, these analyses were derived from a set of heterogeneous studies, with varying levels of adjustments for confounders such as age, height and smoking, which are in themselves also known risk factors for the development of AF and impaired lung function. The attributable fraction of the population with known risk factors has been estimated to be about one-half, indicating that there may be additional risk factors which are not fully elaborated (41–43). Obviously, the regression models could not fully represent the biological complexity of common risk factors affecting lung and heart health.

Consistent with the causal inference on lung function and AF from the newest two sample bidirectional MR study (25), which used UK Biobank summary statistics of FEV1 and FVC and the same AF dataset (including UK Biobank individuals) as ours, we found no evidence of a causal association between FEV1, FVC, and AF. Moreover, consistent results were obtained in the recurring MR analyses. Another MR study (44) found that taller people had higher risk of AF and considering part instruments of the lung function were also related to height, which may account for the invalid results of height adjustment in GWAS summary data. Furthermore, the positive relation of FEV1/FVC with AF in our uvMR Egger analysis is somewhat unexpected although the association disappeared after adjusting for FEV1 and FVC in mvMR method. Observational study (45) indicated that impaired FEV1 and FVC was related to larger left ventricular mass; impaired FEV1/FVC was related to smaller left atrial internal dimension, as well as FEV1 is a measure of severity of airway obstruction while FVC represents overall vital capacity (46). It can be seen that differential pattern of lung function decline reflect distinct effects on cardiopulmonary phenotypes. In terms of genetic mechanisms, the multiple gene regions associated with FEV1 play roles in biological pathways of inflammation, morphogenesis and development; FVC play roles in lung tissue repair, and FEV1/FVC play roles in inflammation and morphogenesis or development (47–49). Thus, we can see that different arguments of lung function may share genetic mechanisms, which could potentially bias estimates of uvMR analyses by violating the horizontal pleiotropy assumption. Our findings successfully accounted for the bias by using mvMR analyses. Although the responsible mechanisms are yet to be defined, inflammation may be the prominently shared risk factors. The correlation between declined FEV1 and FEV1/FVC and the incidence of AF was significantly attenuated after adjusting the inflammation biomarkers in a cohort study (7); FEV1 and FVC were inversely correlated with C-reactive protein (CRP) and fibrinogen in early adulthood (50, 51); the associations were identified between FEV1 and FVC and circulating levels of adhesion molecules such as intercellular adhesion molecule (ICAM) and P-selection (52); indicating that some risks may be mediated by inflammation. In addition, cardiovascular diseases (CVDs) and relevant confounding (18, 19) (e.g., inefficient clearance of cardiotoxic substance or imbalance of ventilation/perfusion ratio) may also bring mediating effect in causal inference between AF and impaired lung function, which need further investigation.

Two-sample MR analysis is a powerful method for causal estimate between exposures and outcome using summary statistic, but we should interpret our findings with caution because of the following limitations. First, although both lung function and AF studies were based on European population with no cohort overlap between the meta-analysis, but might have some sample overlap, which would lead to inflation of Type 1 error rate (53). In addition, the two GWAS we used were both adjusted for age, height, gender and smoking, which may produce collider bias and lead to identification of invalid IVs and thus affect the interpretability and validity of the results (54). Lastly, the results exhibited a lack of evidence from non-European populations.

In conclusion, a causal relationship of impaired lung function with AF was not evident in this study. Even so, it does not hint that improvement of lung function in AF patients and preventing the AF risk in patients with impaired lung function are unnecessary. In addition, lung function and AF were both associated with inflammation, which may be potential pathway, warranting further study. New generations of GWAS with a higher explanatory efficacy and more representative population should be explored and investigated in further researches.

## Data Availability Statement

The datasets presented in this study can be found in online repositories. The names of the repository/repositories and accession number(s) can be found in the article/Supplementary Material.

## Author Contributions

QZ conceived this study, conducted data analysis, and wrote this manuscript. XZ, JieZ, BW, XM, QT, JinZ, MJ, YZ, DZ, LW, and WW contributed to methodological guidance and manuscript revision. YW and BW oversaw the implementation of the statistical and analytical methods and revised and finalized this manuscript. All authors contributed to the article and approved the submitted version.

## Funding

This work was supported by the National Nature Science Foundation of China (NFSC 81673247), a China-Australian collaborative grant (NSFC 81561128020-NHMRC APP1112767), and National Key R&D Program of China (2018YFC2000704).

## Conflict of Interest

The authors declare that the research was conducted in the absence of any commercial or financial relationships that could be construed as a potential conflict of interest.

## Publisher's Note

All claims expressed in this article are solely those of the authors and do not necessarily represent those of their affiliated organizations, or those of the publisher, the editors and the reviewers. Any product that may be evaluated in this article, or claim that may be made by its manufacturer, is not guaranteed or endorsed by the publisher.
