# Supplementary material for: Causal Relationship Between Lung Function and Atrial Fibrillation: A Two Sample Univariable and Multivariable, Bidirectional Mendelian Randomization Study
Source: Front Cardiovasc Med. 2021 Nov 11;8:769198. doi: 10.3389/fcvm.2021.769198 (PMC8635999; doi:10.3389/fcvm.2021.769198)
Supplement: Supplementary file 1 [file Table_1.DOCX]

Supplementary Material

# Supplementary Tables for original results

Supplement Table 1A. SNPs for lung function in the uvMR analyses: Harmonized Data

|  |  |  |  |  |  |  |  |  |  |  |  | Outcome: AF | | |
| --- | --- | --- | --- | --- | --- | --- | --- | --- | --- | --- | --- | --- | --- | --- |
| SNP | Exposure | CHR | POS | EA | OA | EAF | Beta | SE | *P* | R^2^ | F_statistic | Beta | SE | *P* |
| rs10888384 | FEV1 | 1 | 150574256 | T | C | 0.557 | -0.0318 | 0.00553 | 9.39E-09 | 0.000499049 | 39.471016 | -0.0089 | 0.0067 | 0.1833 |
| rs34712979 | FEV1 | 4 | 106819053 | A | G | 0.246 | -0.0664 | 0.00691 | 7.58E-22 | 0.001635583 | 129.50957 | 0.005 | 0.0093 | 0.5911 |
| rs1032296 | FEV1 | 4 | 145434688 | C | T | 0.599 | 0.0377 | 0.00565 | 2.54E-11 | 0.000682785 | 54.013072 | 0.0029 | 0.0068 | 0.6667 |
| rs3995090 | FEV1 | 5 | 147845815 | C | A | 0.407 | 0.0336 | 0.00564 | 2.48E-09 | 0.000544951 | 43.10352 | 0.002 | 0.0067 | 0.768 |
| rs13361953 | FEV1 | 5 | 156926442 | C | T | 0.336 | -0.0367 | 0.00582 | 2.81E-10 | 0.000600993 | 47.538878 | 0.0011 | 0.007 | 0.8773 |
| rs4728570 | FEV1 | 7 | 84728349 | A | G | 0.544 | 0.032 | 0.00562 | 1.22E-08 | 0.000508035 | 40.18211 | 0.0059 | 0.0069 | 0.3896 |
| rs12783223 | FEV1 | 10 | 12244143 | C | T | 0.536 | 0.0334 | 0.00524 | 1.79E-10 | 0.000554888 | 43.889952 | 0.0014 | 0.0067 | 0.8311 |
| rs3858703 | FEV1 | 12 | 863517 | A | G | 0.705 | -0.0324 | 0.00583 | 2.56E-08 | 0.000436648 | 34.533387 | 0.008 | 0.0073 | 0.2763 |
| rs8033889 | FEV1 | 15 | 71680080 | T | G | 0.223 | -0.036 | 0.00626 | 9.07E-09 | 0.000449118 | 35.520112 | 0.0091 | 0.0081 | 0.2598 |
| rs3754512 | FVC | 1 | 17309718 | T | C | 0.513 | 0.0348 | 0.00529 | 4.94E-11 | 0.000605111 | 47.864777 | -0.0114 | 0.0067 | 0.08897 |
| rs10888384 | FVC | 1 | 150574256 | T | C | 0.557 | -0.0334 | 0.00556 | 1.76E-09 | 0.000550531 | 43.545107 | -0.0089 | 0.0067 | 0.1833 |
| rs7574127 | FVC | 2 | 56011107 | T | C | 0.265 | -0.0391 | 0.00628 | 4.87E-10 | 0.000595548 | 47.107906 | 0.0191 | 0.0076 | 0.01221 |
| rs1714518 | FVC | 3 | 158213423 | T | C | 0.505 | 0.031 | 0.00555 | 2.32E-08 | 0.000480452 | 37.999425 | 0.008 | 0.0066 | 0.2299 |
| rs6813090 | FVC | 4 | 89827071 | C | A | 0.772 | 0.0377 | 0.00659 | 1.10E-08 | 0.00050034 | 39.573143 | -0.0074 | 0.008 | 0.3576 |
| rs113881526 | FVC | 4 | 106781636 | G | T | 0.0677 | 0.0619 | 0.0111 | 2.57E-08 | 0.000483677 | 38.254639 | 0.0072 | 0.0137 | 0.5974 |
| rs4371882 | FVC | 6 | 7793169 | G | A | 0.183 | -0.0385 | 0.00679 | 1.36E-08 | 0.000443225 | 35.053831 | 0.0046 | 0.0086 | 0.5958 |
| rs4244844 | FVC | 12 | 28586995 | G | A | 0.442 | -0.0412 | 0.00532 | 9.24E-15 | 0.0008373 | 66.246515 | -0.0087 | 0.0066 | 0.1896 |
| rs3754512 | FEV1/FVC | 1 | 17309718 | T | C | 0.512 | -0.0415 | 0.00524 | 2.33E-15 | 0.000860629 | 68.093907 | -0.0114 | 0.0067 | 0.08897 |
| rs11124193 | FEV1/FVC | 2 | 239875322 | G | A | 0.204 | 0.0445 | 0.00678 | 5.29E-11 | 0.000643122 | 50.873428 | -0.0064 | 0.0083 | 0.4359 |
| rs35536382 | FEV1/FVC | 3 | 25534688 | T | G | 0.0601 | 0.0605 | 0.0111 | 4.66E-08 | 0.00041352 | 32.703546 | 0.0015 | 0.0149 | 0.9219 |
| rs2609280 | FEV1/FVC | 4 | 89854961 | G | A | 0.772 | -0.0457 | 0.00652 | 2.33E-12 | 0.000735215 | 58.163739 | -0.0086 | 0.008 | 0.2857 |
| rs34712979 | FEV1/FVC | 4 | 106819053 | A | G | 0.246 | -0.052 | 0.00686 | 3.43E-14 | 0.001003097 | 79.377488 | 0.005 | 0.0093 | 0.5911 |
| rs13147758 | FEV1/FVC | 4 | 145460230 | G | A | 0.419 | 0.0538 | 0.00555 | 2.78E-22 | 0.001409239 | 111.5618 | 8.00E-04 | 0.0067 | 0.9014 |
| rs10940518 | FEV1/FVC | 5 | 56270794 | T | C | 0.671 | 0.0335 | 0.00583 | 9.45E-09 | 0.000495494 | 39.189672 | 0.0091 | 0.007 | 0.1918 |
| rs6889822 | FEV1/FVC | 5 | 147846707 | G | A | 0.383 | 0.0345 | 0.00573 | 1.72E-09 | 0.000562538 | 44.495373 | 0.0018 | 0.0068 | 0.7981 |
| rs13361953 | FEV1/FVC | 5 | 156926442 | C | T | 0.336 | -0.035 | 0.00578 | 1.45E-09 | 0.000546605 | 43.234381 | 0.0011 | 0.007 | 0.8773 |
| rs2070600 | FEV1/FVC | 6 | 32151443 | T | C | 0.0582 | 0.122 | 0.0122 | 1.78E-23 | 0.001631666 | 129.19892 | -0.0417 | 0.0165 | 0.01173 |
| rs73780219 | FEV1/FVC | 6 | 142722866 | A | G | 0.0274 | 0.14 | 0.0171 | 2.46E-16 | 0.00104465 | 82.669093 | -0.0443 | 0.0201 | 0.02782 |
| rs57062879 | FEV1/FVC | 10 | 12278525 | G | A | 0.515 | 0.0302 | 0.00519 | 5.69E-09 | 0.00045561 | 36.033722 | 0.0033 | 0.0066 | 0.6151 |
| rs2413932 | FEV1/FVC | 15 | 49383481 | T | C | 0.729 | -0.0341 | 0.00588 | 6.76E-09 | 0.000459447 | 36.337372 | 0.0143 | 0.0075 | 0.05767 |
| rs8033889 | FEV1/FVC | 15 | 71680080 | T | G | 0.223 | -0.0568 | 0.00622 | 7.01E-20 | 0.001118028 | 88.482367 | 0.0091 | 0.0081 | 0.2598 |
| rs35420030 | FEV1/FVC | 16 | 53935407 | C | T | 0.0606 | 0.0732 | 0.0117 | 3.63E-10 | 0.000610064 | 48.256822 | 0.015 | 0.0154 | 0.3297 |
| rs4888368 | FEV1/FVC | 16 | 75305331 | A | C | 0.53 | -0.0331 | 0.00563 | 4.00E-09 | 0.000545833 | 43.173294 | -0.0114 | 0.0068 | 0.09312 |

SNP: single-nucleotide polymorphism; CHR: Chromosome; POS: Position; EA: effect allele; OA: other allele; EAF, effect allele frequency; SE: standard error; R^2^: variance for each SNP, R^2^ = 2×EAF× (1-EAF) × Beta^2^; F_statistic = R^2^ × (N-2) / (1-R^2^), N: the number of individuals in the exposure GWAS.

FEV1: forced expiratory volume in one second; FVC: forced vital capacity; FEV1/FVC: the ratio of FEV1 over FVC; AF: atrial fibrillation.

Supplement Table 1B. SNPs for AF in the uvMR analyses: Harmonized Data

|  |  |  |  |  |  |  |  | |  |  |  |  | Outcomes | | |  |  | |  | |  |  |  |  |  |
| --- | --- | --- | --- | --- | --- | --- | --- | --- | --- | --- | --- | --- | --- | --- | --- | --- | --- | --- | --- | --- | --- | --- | --- | --- | --- |
|  |  |  |  |  |  |  |  | |  |  |  |  | FEV1 | | | FVC | | | | | | | FEV1/FVC | | |
| SNP | Exposure | CHR | POS | EA | OA | EAF | Beta | SE | *P* | | R^2^ | F_statistic | Beta | SE | *P* | Beta | | SE | | *P* | | | Beta | SE | *P* |
| rs284277 | AF | 1 | 10790797 | C | A | 0.3826 | 0.0422 | 0.0069 | 1.25E-09 | | 0.00084133 | 868.00207 | -0.00465 | 0.00558 | 0.404 | -0.0023 | | 0.00555 | | 0.679 | | | -0.00085 | 0.00554 | 0.878 |
| rs7529220 | AF | 1 | 22282619 | C | T | 0.8469 | 0.0621 | 0.0098 | 1.98E-10 | | 0.001000047 | 1031.9147 | -0.0048 | 0.00733 | 0.512 | -0.0073 | | 0.00735 | | 0.321 | | | -0.0018 | 0.00729 | 0.805 |
| rs146518726 | AF | 1 | 51535039 | G | A | 0.9672 | -0.1605 | 0.0207 | 8.27E-15 | | 0.001634445 | 1687.5993 | -0.016 | 0.0178 | 0.368 | 8.07E-05 | | 0.0179 | | 0.996 | | | -0.0283 | 0.0177 | 0.109 |
| rs1545300 | AF | 1 | 112464004 | C | T | 0.6909 | 0.0558 | 0.0073 | 1.48E-14 | | 0.00132988 | 1372.7115 | -0.00296 | 0.00601 | 0.622 | 0.000288 | | 0.00603 | | 0.962 | | | -0.00615 | 0.00597 | 0.303 |
| rs6689306 | AF | 1 | 154395946 | G | A | 0.5872 | -0.046 | 0.0068 | 1.36E-11 | | 0.001025821 | 1058.5366 | 0.00137 | 0.00561 | 0.807 | 0.00348 | | 0.00563 | | 0.537 | | | -0.00529 | 0.00558 | 0.343 |
| rs11264280 | AF | 1 | 154862952 | C | T | 0.667 | -0.1347 | 0.0071 | 3.07E-79 | | 0.008060004 | 8376.037 | 0.00366 | 0.00592 | 0.536 | 0.00685 | | 0.00594 | | 0.249 | | | -0.00475 | 0.00587 | 0.419 |
| rs7526113 | AF | 1 | 169768981 | G | A | 0.1491 | 0.0709 | 0.0107 | 3.51E-11 | | 0.001275495 | 1316.5024 | 0.00358 | 0.00883 | 0.685 | 0.00209 | | 0.00886 | | 0.814 | | | -0.00309 | 0.00877 | 0.725 |
| rs591715 | AF | 1 | 170642509 | G | A | 0.8061 | 0.0787 | 0.0084 | 1.02E-20 | | 0.001936182 | 1999.7542 | -0.00614 | 0.00707 | 0.385 | -0.00651 | | 0.00711 | | 0.36 | | | 0.00502 | 0.00702 | 0.475 |
| rs10753933 | AF | 1 | 203026214 | G | T | 0.5518 | -0.0609 | 0.0067 | 9.84E-20 | | 0.001834502 | 1894.5423 | -0.00889 | 0.00557 | 0.111 | -0.0085 | | 0.00559 | | 0.129 | | | 0.00172 | 0.00554 | 0.756 |
| rs7578393 | AF | 2 | 26165528 | C | T | 0.204 | -0.0614 | 0.0088 | 2.42E-12 | | 0.001224362 | 1263.6615 | -0.00552 | 0.00683 | 0.419 | -0.00742 | | 0.00686 | | 0.279 | | | 0.00336 | 0.00679 | 0.621 |
| rs2312555 | AF | 2 | 70040542 | G | A | 0.5399 | 0.0512 | 0.0067 | 1.63E-14 | | 0.001302373 | 1344.2814 | -0.00296 | 0.00552 | 0.592 | -0.00313 | | 0.00554 | | 0.573 | | | 0.000151 | 0.00549 | 0.978 |
| rs72926475 | AF | 2 | 86594487 | G | A | 0.8772 | 0.0683 | 0.0102 | 2.37E-11 | | 0.001005005 | 1037.036 | 0.0112 | 0.00835 | 0.181 | 0.00143 | | 0.00837 | | 0.864 | | | 0.0147 | 0.00829 | 0.0757 |
| rs28387148 | AF | 2 | 127433465 | C | T | 0.8949 | -0.0741 | 0.0113 | 6.25E-11 | | 0.001032865 | 1065.8134 | -0.014 | 0.00981 | 0.154 | -0.0137 | | 0.00985 | | 0.165 | | | 0.000222 | 0.00973 | 0.982 |
| rs17507821 | AF | 2 | 175428639 | C | T | 0.335 | 0.0407 | 0.0071 | 8.75E-09 | | 0.000738049 | 761.36805 | -0.00761 | 0.0059 | 0.197 | -0.00659 | | 0.00592 | | 0.266 | | | -0.00134 | 0.00586 | 0.819 |
| rs2288327 | AF | 2 | 179411665 | G | A | 0.1564 | 0.0919 | 0.0089 | 7.26E-25 | | 0.002228611 | 2302.4596 | -0.0185 | 0.00741 | 0.0123 | -0.0116 | | 0.00743 | | 0.118 | | | -0.0107 | 0.00736 | 0.145 |
| rs3820888 | AF | 2 | 201180023 | C | T | 0.3921 | 0.0684 | 0.0068 | 5.75E-24 | | 0.002230341 | 2304.2502 | -0.00843 | 0.00563 | 0.134 | -0.0121 | | 0.00565 | | 0.0321 | | | 0.00206 | 0.00559 | 0.712 |
| rs7650482 | AF | 3 | 12841804 | G | A | 0.6401 | 0.0711 | 0.007 | 1.79E-24 | | 0.002329158 | 2406.5801 | 0.00165 | 0.00546 | 0.762 | 0.003 | | 0.00548 | | 0.584 | | | -0.00511 | 0.00543 | 0.346 |
| rs73041705 | AF | 3 | 24463235 | C | T | 0.2985 | -0.0443 | 0.0073 | 1.55E-09 | | 0.000821882 | 847.92078 | 0.0024 | 0.00577 | 0.677 | 0.00495 | | 0.00577 | | 0.391 | | | -0.00125 | 0.00574 | 0.827 |
| rs34080181 | AF | 3 | 66454191 | G | A | 0.621 | 0.0446 | 0.0069 | 1.28E-10 | | 0.000936333 | 966.10892 | 0.00209 | 0.0057 | 0.714 | -0.0023 | | 0.00571 | | 0.687 | | | 0.0111 | 0.00566 | 0.0507 |
| rs9868990 | AF | 3 | 69413694 | C | T | 0.6573 | -0.0413 | 0.0072 | 7.91E-09 | | 0.000768436 | 792.73937 | 0.00935 | 0.00598 | 0.118 | 0.00882 | | 0.006 | | 0.142 | | | 0.00618 | 0.00593 | 0.297 |
| rs10804493 | AF | 3 | 111554426 | G | A | 0.3495 | -0.0558 | 0.007 | 1.63E-15 | | 0.001415771 | 1461.4936 | 0.00566 | 0.00582 | 0.331 | 4.53E-05 | | 0.00585 | | 0.994 | | | 0.0092 | 0.00579 | 0.112 |
| rs1278493 | AF | 3 | 135814009 | G | A | 0.4355 | 0.0389 | 0.0068 | 8.77E-09 | | 0.000744014 | 767.52632 | -0.00381 | 0.00555 | 0.492 | -0.00481 | | 0.00557 | | 0.387 | | | 0.00218 | 0.00551 | 0.692 |
| rs7612445 | AF | 3 | 179172979 | G | T | 0.8121 | -0.0493 | 0.0084 | 4.81E-09 | | 0.000741754 | 765.19323 | -0.00533 | 0.00719 | 0.459 | -0.00342 | | 0.00722 | | 0.636 | | | -0.00676 | 0.00715 | 0.344 |
| rs60902112 | AF | 3 | 194800853 | C | T | 0.7738 | -0.0445 | 0.0079 | 1.72E-08 | | 0.00069322 | 715.09089 | -0.00446 | 0.00676 | 0.509 | -0.0113 | | 0.00678 | | 0.0962 | | | 0.0136 | 0.00672 | 0.0423 |
| rs1458038 | AF | 4 | 81164723 | C | T | 0.6913 | -0.0434 | 0.0072 | 1.74E-09 | | 0.00080392 | 829.37445 | 0.00365 | 0.00603 | 0.545 | 0.00363 | | 0.00613 | | 0.553 | | | 0.000557 | 0.00599 | 0.926 |
| rs13121747 | AF | 4 | 111514751 | G | A | 0.6483 | 0.0516 | 0.0077 | 1.87E-11 | | 0.001214165 | 1253.1243 | 0.00484 | 0.00647 | 0.455 | 0.00269 | | 0.00649 | | 0.678 | | | 0.00489 | 0.00642 | 0.446 |
| rs6854883 | AF | 4 | 111607315 | C | T | 0.8501 | -0.2822 | 0.0093 | 5.65E-203 | | 0.020296243 | 21355.494 | -0.00211 | 0.00771 | 0.784 | 0.000395 | | 0.00775 | | 0.959 | | | -0.00833 | 0.00766 | 0.277 |
| rs75310513 | AF | 4 | 111765077 | C | T | 0.7963 | 0.1418 | 0.0084 | 2.49E-63 | | 0.006523042 | 6768.324 | -0.0005 | 0.00674 | 0.941 | 0.00141 | | 0.00677 | | 0.835 | | | 0.00151 | 0.0067 | 0.822 |
| rs10520260 | AF | 4 | 174447349 | G | A | 0.3214 | -0.0457 | 0.0073 | 3.36E-10 | | 0.000911008 | 939.95418 | -0.00743 | 0.00597 | 0.213 | -0.00574 | | 0.006 | | 0.338 | | | 0.00192 | 0.00593 | 0.746 |
| rs74500426 | AF | 4 | 174642789 | G | T | 0.9236 | 0.0921 | 0.0127 | 4.29E-13 | | 0.001197089 | 1235.4793 | 0.00348 | 0.00977 | 0.722 | 0.00197 | | 0.0098 | | 0.841 | | | -0.00075 | 0.0097 | 0.939 |
| rs337705 | AF | 5 | 113737062 | G | T | 0.3749 | 0.0564 | 0.0068 | 1.63E-16 | | 0.001490916 | 1539.1816 | 0.0046 | 0.00563 | 0.414 | 0.00423 | | 0.00566 | | 0.455 | | | -0.00133 | 0.0056 | 0.812 |
| rs1446074 | AF | 5 | 114445023 | C | T | 0.507 | -0.0385 | 0.0067 | 9.48E-09 | | 0.00074098 | 764.39351 | -0.0038 | 0.00556 | 0.495 | 2.81E-05 | | 0.00558 | | 0.996 | | | -0.00657 | 0.00552 | 0.234 |
| rs2040862 | AF | 5 | 137419989 | C | T | 0.8225 | -0.1084 | 0.0087 | 1.08E-35 | | 0.003431017 | 3548.9852 | -0.0161 | 0.00708 | 0.023 | -0.00596 | | 0.0071 | | 0.402 | | | -0.0157 | 0.00703 | 0.0254 |
| rs6580277 | AF | 5 | 142818123 | G | A | 0.2369 | 0.067 | 0.0079 | 1.64E-17 | | 0.001623028 | 1675.7927 | -0.00964 | 0.00654 | 0.14 | -0.00818 | | 0.00656 | | 0.212 | | | -0.00495 | 0.00649 | 0.446 |
| rs6891790 | AF | 5 | 172670745 | G | T | 0.7172 | 0.0729 | 0.0076 | 4.53E-22 | | 0.002155781 | 2227.0539 | -0.0123 | 0.00616 | 0.0466 | -0.00457 | | 0.00618 | | 0.46 | | | -0.00711 | 0.00612 | 0.245 |
| rs73366713 | AF | 6 | 16415751 | G | A | 0.8604 | 0.1035 | 0.0099 | 1.53E-25 | | 0.002573336 | 2659.5262 | -0.0105 | 0.00781 | 0.18 | -0.00352 | | 0.00783 | | 0.653 | | | -0.014 | 0.00775 | 0.0702 |
| rs4712329 | AF | 6 | 18235776 | G | A | 0.7298 | -0.0643 | 0.0075 | 8.43E-18 | | 0.001630576 | 1683.5988 | -0.00187 | 0.00586 | 0.75 | -0.0003 | | 0.00588 | | 0.96 | | | -0.00125 | 0.00583 | 0.83 |
| rs3176326 | AF | 6 | 36647289 | G | A | 0.8018 | 0.0626 | 0.0085 | 1.42E-13 | | 0.001245513 | 1285.5186 | 0.0105 | 0.00671 | 0.116 | 0.00312 | | 0.00673 | | 0.643 | | | 0.0102 | 0.00667 | 0.126 |
| rs2031522 | AF | 6 | 87821501 | G | A | 0.3764 | -0.0436 | 0.0068 | 1.47E-10 | | 0.000892398 | 920.73609 | -0.00535 | 0.00567 | 0.346 | -0.00217 | | 0.0057 | | 0.704 | | | -0.00476 | 0.00564 | 0.398 |
| rs13195459 | AF | 6 | 122403559 | G | A | 0.6385 | 0.0623 | 0.007 | 4.15E-19 | | 0.001791741 | 1850.3031 | -0.00743 | 0.00597 | 0.213 | -0.0043 | | 0.00584 | | 0.461 | | | 0.00738 | 0.00578 | 0.202 |
| rs117984853 | AF | 6 | 149399100 | G | T | 0.8987 | -0.1228 | 0.012 | 1.34E-24 | | 0.002745686 | 2838.1394 | 0.00348 | 0.00977 | 0.722 | 0.00236 | | 0.01 | | 0.814 | | | 0.00933 | 0.0099 | 0.346 |
| rs55734480 | AF | 7 | 14372009 | G | A | 0.7506 | -0.0548 | 0.0078 | 2.20E-12 | | 0.001124336 | 1160.3084 | 0.0046 | 0.00563 | 0.414 | -0.00788 | | 0.00611 | | 0.197 | | | -0.00356 | 0.00605 | 0.556 |
| rs6462079 | AF | 7 | 28415827 | G | A | 0.2792 | -0.0466 | 0.0076 | 8.79E-10 | | 0.000874041 | 901.77982 | -0.0038 | 0.00556 | 0.495 | -0.00609 | | 0.00612 | | 0.319 | | | 0.00118 | 0.00606 | 0.846 |
| rs35005436 | AF | 7 | 74134911 | C | T | 0.1551 | 0.0612 | 0.0097 | 3.34E-10 | | 0.000981635 | 1012.8968 | -0.0161 | 0.00708 | 0.023 | -0.00295 | | 0.00812 | | 0.717 | | | 0.000336 | 0.00804 | 0.967 |
| rs56201652 | AF | 7 | 92278116 | G | A | 0.733 | 0.0531 | 0.0075 | 1.74E-12 | | 0.001103657 | 1138.9446 | -0.00964 | 0.00654 | 0.14 | -0.00129 | | 0.00624 | | 0.836 | | | 0.00662 | 0.00617 | 0.283 |
| rs11773845 | AF | 7 | 116191301 | C | A | 0.4144 | -0.1054 | 0.0067 | 2.39E-55 | | 0.005391778 | 5588.1586 | -0.0123 | 0.00616 | 0.0466 | 0.00707 | | 0.00562 | | 0.209 | | | -0.00166 | 0.0056 | 0.767 |
| rs55985730 | AF | 7 | 128417044 | G | T | 0.06 | 0.0867 | 0.0149 | 5.24E-09 | | 0.000847905 | 874.79124 | -0.0105 | 0.00781 | 0.18 | 0.0056 | | 0.0124 | | 0.65 | | | -0.028 | 0.0122 | 0.0219 |
| rs7789146 | AF | 7 | 150661409 | G | A | 0.8213 | 0.0584 | 0.0087 | 2.12E-11 | | 0.001001111 | 1033.013 | -0.00187 | 0.00586 | 0.75 | -0.00592 | | 0.00718 | | 0.41 | | | 0.00232 | 0.0071 | 0.744 |
| rs7508 | AF | 8 | 17913970 | G | A | 0.2891 | -0.0711 | 0.0075 | 1.69E-21 | | 0.002077906 | 2146.4358 | 0.0105 | 0.00671 | 0.116 | 0.00231 | | 0.00582 | | 0.691 | | | 0.00868 | 0.00576 | 0.132 |
| rs62521286 | AF | 8 | 124551975 | G | A | 0.0663 | 0.1202 | 0.0135 | 4.50E-19 | | 0.001788792 | 1847.2519 | -0.00535 | 0.00567 | 0.346 | -0.013 | | 0.0111 | | 0.245 | | | 0.0202 | 0.011 | 0.0669 |
| rs6994744 | AF | 8 | 141740868 | C | A | 0.4954 | 0.0405 | 0.0066 | 1.10E-09 | | 0.000820056 | 846.03497 | -0.00743 | 0.00597 | 0.213 | -0.00959 | | 0.00557 | | 0.0854 | | | 0.00662 | 0.00552 | 0.23 |
| rs10821415 | AF | 9 | 97713459 | C | A | 0.5868 | -0.0821 | 0.0067 | 2.92E-34 | | 0.003268637 | 3380.472 | 0.00348 | 0.00977 | 0.722 | 0.00179 | | 0.00567 | | 0.752 | | | -0.0042 | 0.00561 | 0.454 |
| rs2274115 | AF | 9 | 139094773 | G | A | 0.7003 | 0.0487 | 0.0076 | 1.69E-10 | | 0.00099554 | 1027.2593 | 0.0046 | 0.00563 | 0.414 | -0.0142 | | 0.00648 | | 0.0283 | | | 0.00362 | 0.0064 | 0.572 |
| rs12245149 | AF | 10 | 65321147 | C | A | 0.5261 | 0.047 | 0.0067 | 1.66E-12 | | 0.00110149 | 1136.7058 | -0.0038 | 0.00556 | 0.495 | 0.00236 | | 0.00552 | | 0.669 | | | 0.00492 | 0.0055 | 0.371 |
| rs4746374 | AF | 10 | 77942661 | G | A | 0.1688 | 0.0539 | 0.0088 | 1.10E-09 | | 0.00081524 | 841.06282 | -0.00344 | 0.00746 | 0.644 | -0.00482 | | 0.00748 | | 0.52 | | | 0.00187 | 0.00741 | 0.801 |
| rs11598047 | AF | 10 | 105342672 | G | A | 0.1621 | 0.1537 | 0.009 | 8.95E-66 | | 0.006417309 | 6657.9059 | -0.00158 | 0.0079 | 0.841 | -0.00344 | | 0.00793 | | 0.664 | | | -0.00192 | 0.00785 | 0.807 |
| rs35696766 | AF | 10 | 105536662 | G | T | 0.8849 | -0.131 | 0.0103 | 9.14E-37 | | 0.003495764 | 3616.1937 | -0.00991 | 0.00867 | 0.253 | -0.013 | | 0.00871 | | 0.136 | | | 0.015 | 0.00862 | 0.0818 |
| rs10741807 | AF | 11 | 20011445 | C | T | 0.7551 | -0.0729 | 0.0079 | 1.59E-20 | | 0.001965524 | 2030.119 | -0.00619 | 0.00628 | 0.324 | -0.00578 | | 0.0063 | | 0.359 | | | 0.00193 | 0.00624 | 0.757 |
| rs7115531 | AF | 11 | 121604722 | G | A | 0.7513 | -0.044 | 0.0078 | 2.00E-08 | | 0.000723477 | 746.32428 | 4.92E-05 | 0.00645 | 0.994 | 0.00752 | | 0.00649 | | 0.247 | | | -0.0102 | 0.00641 | 0.111 |
| rs76097649 | AF | 11 | 128764570 | G | A | 0.9067 | -0.1151 | 0.0124 | 1.26E-20 | | 0.002241434 | 2315.7367 | -0.0109 | 0.0103 | 0.288 | -0.00422 | | 0.0103 | | 0.683 | | | -0.0102 | 0.0102 | 0.318 |
| rs4963776 | AF | 12 | 24779491 | G | T | 0.8179 | 0.0913 | 0.0088 | 1.84E-25 | | 0.002483029 | 2565.9616 | 0.00539 | 0.00687 | 0.433 | 0.000686 | | 0.00689 | | 0.921 | | | 0.00507 | 0.00683 | 0.458 |
| rs2860482 | AF | 12 | 57105938 | C | A | 0.726 | -0.054 | 0.0076 | 1.21E-12 | | 0.001160125 | 1197.2851 | 0.00819 | 0.00631 | 0.194 | 0.014 | | 0.00634 | | 0.0273 | | | -0.00866 | 0.00627 | 0.167 |
| rs71454237 | AF | 12 | 70013415 | G | A | 0.791 | 0.062 | 0.0084 | 1.78E-13 | | 0.001270972 | 1311.8289 | -0.00209 | 0.00701 | 0.766 | -0.00174 | | 0.00704 | | 0.805 | | | -0.00206 | 0.00696 | 0.767 |
| rs775498 | AF | 12 | 70071513 | G | A | 0.2798 | 0.0423 | 0.0074 | 1.05E-08 | | 0.000721127 | 743.89833 | 0.0023 | 0.00609 | 0.706 | -0.00562 | | 0.00612 | | 0.358 | | | 0.00944 | 0.00605 | 0.119 |
| rs1565765 | AF | 12 | 76238855 | C | T | 0.4723 | 0.0388 | 0.0067 | 6.10E-09 | | 0.00075041 | 774.12883 | -0.00511 | 0.00548 | 0.351 | -0.00567 | | 0.00551 | | 0.303 | | | -0.00048 | 0.00544 | 0.929 |
| rs883079 | AF | 12 | 114793240 | C | T | 0.2926 | -0.0981 | 0.0074 | 2.84E-40 | | 0.00398389 | 4123.1559 | -0.00715 | 0.00614 | 0.244 | -0.00611 | | 0.00616 | | 0.321 | | | -0.00049 | 0.0061 | 0.936 |
| rs6560886 | AF | 12 | 133150210 | C | T | 0.7884 | 0.051 | 0.009 | 1.49E-08 | | 0.000867826 | 895.3615 | 0.00306 | 0.00737 | 0.678 | 0.00444 | | 0.00739 | | 0.548 | | | -0.00208 | 0.00731 | 0.776 |
| rs9506925 | AF | 13 | 23368943 | C | T | 0.7331 | -0.0449 | 0.0075 | 2.72E-09 | | 0.000788923 | 813.89047 | 0.019 | 0.00591 | 0.0013 | 0.017 | | 0.00592 | | 0.00411 | | | 0.00212 | 0.00587 | 0.719 |
| rs35569628 | AF | 13 | 113872712 | C | T | 0.223 | -0.0452 | 0.008 | 1.38E-08 | | 0.000707999 | 730.3467 | 0.00923 | 0.00652 | 0.157 | 0.0202 | | 0.00654 | | 0.00207 | | | -0.0174 | 0.00647 | 0.00736 |
| rs28631169 | AF | 14 | 23888183 | C | T | 0.8018 | -0.0522 | 0.0084 | 5.35E-10 | | 0.000866045 | 893.52297 | -0.00591 | 0.00677 | 0.382 | -0.00614 | | 0.00675 | | 0.363 | | | -0.00302 | 0.00668 | 0.651 |
| rs11156751 | AF | 14 | 32990437 | C | T | 0.2853 | 0.0719 | 0.0077 | 6.94E-21 | | 0.002108207 | 2177.8031 | 0.00192 | 0.00601 | 0.749 | -0.00219 | | 0.00603 | | 0.717 | | | 0.00111 | 0.00594 | 0.852 |
| rs10141892 | AF | 14 | 35184323 | C | T | 0.5833 | -0.0452 | 0.0068 | 2.95E-11 | | 0.000993167 | 1024.8083 | -0.00471 | 0.00536 | 0.38 | -0.00466 | | 0.00537 | | 0.385 | | | -0.0005 | 0.00532 | 0.926 |
| rs2738413 | AF | 14 | 64679960 | G | A | 0.5049 | -0.0778 | 0.0067 | 2.55E-31 | | 0.003026129 | 3128.9055 | -0.00019 | 0.00525 | 0.971 | -0.00449 | | 0.00527 | | 0.394 | | | 0.00908 | 0.00522 | 0.0819 |
| rs74884082 | AF | 14 | 73249419 | C | T | 0.7505 | 0.0493 | 0.0078 | 3.48E-10 | | 0.000910217 | 939.13775 | -0.0123 | 0.00666 | 0.0648 | -0.0033 | | 0.00669 | | 0.621 | | | -0.0158 | 0.00662 | 0.017 |
| rs74022964 | AF | 15 | 73677264 | C | T | 0.843 | -0.1132 | 0.009 | 3.51E-36 | | 0.003391955 | 3508.443 | -0.00338 | 0.00707 | 0.633 | -0.00887 | | 0.0071 | | 0.212 | | | 0.00828 | 0.00704 | 0.239 |
| rs140185678 | AF | 16 | 2003016 | G | A | 0.9649 | -0.1659 | 0.0218 | 2.43E-14 | | 0.001864285 | 1925.3573 | -0.0152 | 0.0178 | 0.395 | -0.0346 | | 0.018 | | 0.0541 | | | 0.0318 | 0.0177 | 0.0728 |
| rs7225165 | AF | 17 | 1309850 | G | A | 0.8867 | 0.0655 | 0.0111 | 3.20E-09 | | 0.000862024 | 889.37001 | 0.00387 | 0.00861 | 0.653 | 0.00428 | | 0.00863 | | 0.62 | | | -0.00016 | 0.00855 | 0.985 |
| rs1563304 | AF | 17 | 44874453 | C | T | 0.822 | -0.0644 | 0.0092 | 2.56E-12 | | 0.00121365 | 1252.5922 | -0.0157 | 0.00745 | 0.0355 | -0.0158 | | 0.00748 | | 0.0351 | | | -0.00283 | 0.00741 | 0.702 |
| rs12604076 | AF | 17 | 76773638 | C | T | 0.5223 | -0.0365 | 0.0066 | 3.63E-08 | | 0.0006648 | 685.7543 | 0.000478 | 0.00554 | 0.931 | 0.00885 | | 0.00557 | | 0.112 | | | -0.00813 | 0.00551 | 0.14 |
| rs9953366 | AF | 18 | 46474192 | C | T | 0.6631 | 0.049 | 0.0073 | 1.82E-11 | | 0.001072759 | 1107.0241 | 0.00495 | 0.00569 | 0.384 | 0.00903 | | 0.0057 | | 0.113 | | | -0.00247 | 0.00564 | 0.661 |
| rs2834618 | AF | 21 | 36119111 | G | T | 0.1056 | -0.0944 | 0.0112 | 3.41E-17 | | 0.001683332 | 1738.1614 | -0.00471 | 0.00878 | 0.592 | -0.00903 | | 0.0088 | | 0.305 | | | 0.0057 | 0.00872 | 0.513 |
| rs464901 | AF | 22 | 18597502 | C | T | 0.3353 | -0.0508 | 0.0072 | 1.53E-12 | | 0.001150315 | 1187.1491 | -0.00027 | 0.00553 | 0.961 | -0.00118 | | 0.00554 | | 0.832 | | | -0.00237 | 0.0055 | 0.667 |

SNP: single-nucleotide polymorphism; CHR: Chromosome; POS: Position; EA: effect allele; OA: other allele; EAF, effect allele frequency; SE: standard error; R^2^: variance for each SNP, R^2^ = 2×EAF× (1-EAF) × Beta^2^; F_statistic = R^2^ × (N-2) / (1-R^2^), N: the number of individuals in the exposure GWAS.

FEV1: forced expiratory volume in one second; FVC: forced vital capacity; FEV1/FVC: the ratio of FEV1 over FVC; AF: atrial fibrillation.

Supplement Table 1C. SNPs (28) for lung function in the mvMR analyses: Harmonized Data

|  |  |  |  |  |  |  |  |  |  |  |  | Outcome: AF | | |
| --- | --- | --- | --- | --- | --- | --- | --- | --- | --- | --- | --- | --- | --- | --- |
| SNP | Exposure | CHR | POS | EA | OA | EAF | Beta | SE | *P* | R^2^ | F_statistic | Beta | SE | *P* |
| rs34712979 | FEV1 | 4 | 106819053 | A | G | 0.246 | -0.0664 | 0.00691 | 7.58E-22 | 0.001635583 | 129.5095724 | 0.005 | 0.0093 | 0.5911 |
| rs1032296 | FEV1 | 4 | 145434688 | C | T | 0.599 | 0.0377 | 0.00565 | 2.54E-11 | 0.000682785 | 54.01307191 | 0.0029 | 0.0068 | 0.6667 |
| rs3995090 | FEV1 | 5 | 147845815 | C | A | 0.407 | 0.0336 | 0.00564 | 2.48E-09 | 0.000544951 | 43.10352048 | 0.002 | 0.0067 | 0.768 |
| rs13361953 | FEV1 | 5 | 156926442 | C | T | 0.336 | -0.0367 | 0.00582 | 2.81E-10 | 0.000600993 | 47.53887753 | 0.0011 | 0.007 | 0.8773 |
| rs4728570 | FEV1 | 7 | 84728349 | A | G | 0.544 | 0.032 | 0.00562 | 1.22E-08 | 0.000508035 | 40.18211047 | 0.0059 | 0.0069 | 0.3896 |
| rs12783223 | FEV1 | 10 | 12244143 | C | T | 0.536 | 0.0334 | 0.00524 | 1.79E-10 | 0.000554888 | 43.88995213 | 0.0014 | 0.0067 | 0.8311 |
| rs3858703 | FEV1 | 12 | 863517 | A | G | 0.705 | -0.0324 | 0.00583 | 2.56E-08 | 0.000436648 | 34.53338734 | 0.008 | 0.0073 | 0.2763 |
| rs10888384 | FVC | 1 | 150574256 | T | C | 0.557 | -0.0334 | 0.00556 | 1.76E-09 | 0.000550531 | 43.54510728 | -0.0089 | 0.0067 | 0.1833 |
| rs7574127 | FVC | 2 | 56011107 | T | C | 0.265 | -0.0391 | 0.00628 | 4.87E-10 | 0.000595548 | 47.10790596 | 0.0191 | 0.0076 | 0.01221 |
| rs1714518 | FVC | 3 | 158213423 | T | C | 0.505 | 0.031 | 0.00555 | 2.32E-08 | 0.000480452 | 37.9994249 | 0.008 | 0.0066 | 0.2299 |
| rs6813090 | FVC | 4 | 89827071 | C | A | 0.772 | 0.0377 | 0.00659 | 1.10E-08 | 0.00050034 | 39.57314335 | -0.0074 | 0.008 | 0.3576 |
| rs113881526 | FVC | 4 | 106781636 | G | T | 0.0677 | 0.0619 | 0.0111 | 2.57E-08 | 0.000483677 | 38.25463931 | 0.0072 | 0.0137 | 0.5974 |
| rs4371882 | FVC | 6 | 7793169 | G | A | 0.183 | -0.0385 | 0.00679 | 1.36E-08 | 0.000443225 | 35.05383109 | 0.0046 | 0.0086 | 0.5958 |
| rs4244844 | FVC | 12 | 28586995 | G | A | 0.442 | -0.0412 | 0.00532 | 9.24E-15 | 0.0008373 | 66.24651533 | -0.0087 | 0.0066 | 0.1896 |
| rs3754512 | FEV1/FVC | 1 | 17309718 | T | C | 0.512 | -0.0415 | 0.00524 | 2.33E-15 | 0.000860629 | 68.0939073 | -0.0114 | 0.0067 | 0.08897 |
| rs11124193 | FEV1/FVC | 2 | 239875322 | G | A | 0.204 | 0.0445 | 0.00678 | 5.29E-11 | 0.000643122 | 50.873428 | -0.0064 | 0.0083 | 0.4359 |
| rs35536382 | FEV1/FVC | 3 | 25534688 | T | G | 0.0601 | 0.0605 | 0.0111 | 4.66E-08 | 0.00041352 | 32.70354629 | 0.0015 | 0.0149 | 0.9219 |
| rs2609280 | FEV1/FVC | 4 | 89854961 | G | A | 0.772 | -0.0457 | 0.00652 | 2.33E-12 | 0.000735215 | 58.16373891 | -0.0086 | 0.008 | 0.2857 |
| rs13147758 | FEV1/FVC | 4 | 145460230 | G | A | 0.419 | 0.0538 | 0.00555 | 2.78E-22 | 0.001409239 | 111.5618004 | 8E-04 | 0.0067 | 0.9014 |
| rs10940518 | FEV1/FVC | 5 | 56270794 | T | C | 0.671 | 0.0335 | 0.00583 | 9.45E-09 | 0.000495494 | 39.18967185 | 0.0091 | 0.007 | 0.1918 |
| rs6889822 | FEV1/FVC | 5 | 147846707 | G | A | 0.383 | 0.0345 | 0.00573 | 1.72E-09 | 0.000562538 | 44.49537339 | 0.0018 | 0.0068 | 0.7981 |
| rs2070600 | FEV1/FVC | 6 | 32151443 | T | C | 0.0582 | 0.122 | 0.0122 | 1.78E-23 | 0.001631666 | 129.1989208 | -0.0417 | 0.0165 | 0.01173 |
| rs73780219 | FEV1/FVC | 6 | 142722866 | A | G | 0.0274 | 0.14 | 0.0171 | 2.46E-16 | 0.00104465 | 82.66909318 | -0.0443 | 0.0201 | 0.02782 |
| rs57062879 | FEV1/FVC | 10 | 12278525 | G | A | 0.515 | 0.0302 | 0.00519 | 5.69E-09 | 0.00045561 | 36.03372159 | 0.0033 | 0.0066 | 0.6151 |
| rs2413932 | FEV1/FVC | 15 | 49383481 | T | C | 0.729 | -0.0341 | 0.00588 | 6.76E-09 | 0.000459447 | 36.33737157 | 0.0143 | 0.0075 | 0.05767 |
| rs8033889 | FEV1/FVC | 15 | 71680080 | T | G | 0.223 | -0.0568 | 0.00622 | 7.01E-20 | 0.001118028 | 88.4823665 | 0.0091 | 0.0081 | 0.2598 |
| rs35420030 | FEV1/FVC | 16 | 53935407 | C | T | 0.0606 | 0.0732 | 0.0117 | 3.63E-10 | 0.000610064 | 48.25682246 | 0.015 | 0.0154 | 0.3297 |
| rs4888368 | FEV1/FVC | 16 | 75305331 | A | C | 0.53 | -0.0331 | 0.00563 | 4.00E-09 | 0.000545833 | 43.17329381 | -0.0114 | 0.0068 | 0.09312 |
| rs10888384 | FEV1 | 1 | 150574256 | T | C | 0.557 | -0.0318 | 0.00553 | 9.39E-09 | 0.000499049 | 39.4710156 | - | - | - |
| rs8033889 | FEV1 | 15 | 71680080 | T | G | 0.223 | -0.036 | 0.00626 | 9.07E-09 | 0.000449118 | 35.52011214 | - | - | - |
| rs3754512 | FVC | 1 | 17309718 | T | C | 0.513 | 0.0348 | 0.00529 | 4.94E-11 | 0.000605111 | 47.86477716 | - | - | - |
| rs34712979 | FEV1/FVC | 4 | 106819053 | A | G | 0.246 | -0.052 | 0.00686 | 3.43E-14 | 0.001003097 | 79.37748781 | - | - | - |
| rs13361953 | FEV1/FVC | 5 | 156926442 | C | T | 0.336 | -0.035 | 0.00578 | 1.45E-09 | 0.000546605 | 43.23438137 | - | - | - |

SNP: single-nucleotide polymorphism; CHR: Chromosome; POS: Position; EA: effect allele; OA: other allele; EAF, effect allele frequency; SE: standard error; R^2^: variance for each SNP, R^2^ = 2×EAF× (1-EAF) × Beta^2^; F_statistic = R^2^ × (N-2) / (1-R^2^), N: the number of individuals in the exposure GWAS.

FEV1: forced expiratory volume in one second; FVC: forced vital capacity; FEV1/FVC: the ratio of FEV1 over FVC; AF: atrial fibrillation.

The red font represents the removed repeated SNPs which show larger *P* values.

Supplement Table 2. Forward causal relations of FEV1, FVC and FEV1/FVC with AF performed by uvMR analyses

| Exposure | Methods | Number of SNP | OR | OR_lci95 | OR_uci95 | *P*_value | Q_pval | *I^2^* |
| --- | --- | --- | --- | --- | --- | --- | --- | --- |
| FEV1 |  |  |  |  |  |  |  |  |
|  | IVW | 9 | 1.031 | 0.909 | 1.169 | 0.630 | 0.719 | 0 |
|  | MR Egger | 9 | 1.223 | 0.694 | 2.153 | 0.509 | 0.661 | 0 |
|  | Weighted median | 9 | 1.067 | 0.905 | 1.258 | 0.442 |  |  |
|  | MR-PRESSO (Global test) |  |  |  |  | 0.242 |  |  |
|  | Raw |  |  |  |  | 0.776 |  |  |
|  | Outlier-corrected |  |  |  |  | NA |  |  |
| FVC |  |  |  |  |  |  |  |  |
| Outliers were not removed | IVW | 8 | 1.068 | 0.874 | 1.305 | 0.519 | 0.039 | 52% |
|  | MR Egger | 8 | 2.009 | 0.565 | 7.142 | 0.322 | 0.049 | 53% |
|  | Weighted median | 8 | 1.121 | 0.903 | 1.391 | 0.299 |  |  |
|  | MR-PRESSO (Global test) |  |  |  |  | 0.010 |  |  |
|  | Raw |  |  |  |  | 0.881 |  |  |
|  | Outlier-corrected |  |  |  |  | 0.421 |  |  |
| FVC |  |  |  |  |  |  |  |  |
| Outliers were removed | IVW | 7 | 1.002 | 0.834 | 1.204 | 0.982 | 0.158 | 35% |
|  | MR Egger | 7 | 1.783 | 0.604 | 5.266 | 0.343 | 0.181 | 34% |
|  | Weighted median | 7 | 0.981 | 0.807 | 1.192 | 0.844 |  |  |
|  | MR-PRESSO (Global test) |  |  |  |  | 0.080 |  |  |
|  | Raw |  |  |  |  | 0.421 |  |  |
|  | Outlier-corrected |  |  |  |  | NA |  |  |
| FEV1/FVC |  |  |  |  |  |  |  |  |
|  | IVW | 16 | 1.076 | 0.966 | 1.199 | 0.182 | 0.064 | 38% |
|  | MR Egger | 16 | 1.502 | 1.178 | 1.915 | 0.006 | 0.372 | 7% |
|  | Weighted median | 16 | 1.099 | 0.969 | 1.246 | 0.139 |  |  |
|  | MR-PRESSO (Global test) |  |  |  |  | 0.007 |  |  |
|  | Raw |  |  |  |  | 0.986 |  |  |
|  | Outlier-corrected |  |  |  |  | NA |  |  |

Q_pval: *P* value of the Cochran Q statistic; *I^2^* = (Q-df)/Q×100%; *P* < 0.017 were considered statistically significant.

FEV1: forced expiratory volume in one second; FVC: forced vital capacity; FEV1/FVC: the ratio of FEV1 over FVC; AF: atrial fibrillation.

IVW: inverse-variance weighted; uvMR: univariable mendelian randomization; MR-PRESSO: Pleiotropy Residual Sum and Outlier; OR: odds ratio; SNP: single-nucleotide polymorphism.

Supplement Table 3. Pleiotropic tests for the selected instrumental variables (lung function)

| Exposure | Egger_intercept | SE | *P* value |
| --- | --- | --- | --- |
| FEV1 | -0.006 | 0.011 | 0.565 |
| FVC (outliers were not removed) | -0.024 | 0.024 | 0.361 |
| FVC (outliers were removed) | -0.022 | 0.021 | 0.339 |
| FEV1/FVC | -0.017 | 0.006 | 0.012 |

FEV1: forced expiratory volume in one second; FVC: forced vital capacity; FEV1/FVC: the ratio of FEV1 over FVC; AF: atrial fibrillation; SE: standard error.

Supplement Table 4. Reverse causal relations of AF with FEV1, FVC and FEV1/FVC performed by uvMR analyses

| Outcome | Methods | Number of SNP | OR | OR_lci95 | OR_uci95 | *P*_value | Q_pval | *I^2^* |
| --- | --- | --- | --- | --- | --- | --- | --- | --- |
| FEV1 |  |  |  |  |  |  |  |  |
|  | IVW | 82 | 0.986 | 0.966 | 1.007 | 0.187 | 0.213 | 11% |
|  | MR Egger | 82 | 0.979 | 0.936 | 1.026 | 0.384 | 0.194 | 12% |
|  | Weighted median | 82 | 0.992 | 0.959 | 1.026 | 0.653 |  |  |
|  | MR-PRESSO (Global test) |  |  |  |  | 0.076 |  |  |
|  | Raw |  |  |  |  | 0.690 |  |  |
|  | Outlier-corrected |  |  |  |  | NA |  |  |
| FVC |  |  |  |  |  |  |  |  |
|  | IVW | 82 | 0.985 | 0.965 | 1.006 | 0.158 | 0.162 | 13% |
|  | MR Egger | 82 | 1.002 | 0.957 | 1.050 | 0.922 | 0.157 | 14% |
|  | Weighted median | 82 | 1.001 | 0.969 | 1.034 | 0.954 |  |  |
|  | MR-PRESSO (Global test) |  |  |  |  | 0.084 |  |  |
|  | Raw |  |  |  |  | 0.239 |  |  |
|  | Outlier-corrected |  |  |  |  | NA |  |  |
| FEV1/FVC |  |  |  |  |  |  |  |  |
|  | IVW | 82 | 0.994 | 0.973 | 1.015 | 0.545 | 0.127 | 15% |
|  | MR Egger | 82 | 0.967 | 0.923 | 1.013 | 0.157 | 0.140 | 15% |
|  | Weighted median | 82 | 0.975 | 0.943 | 1.007 | 0.123 |  |  |
|  | MR-PRESSO (Global test) |  |  |  |  | 0.190 |  |  |
|  | Raw |  |  |  |  | 0.484 |  |  |
|  | Outlier-corrected |  |  |  |  | NA |  |  |

Q_pval: *P* value of the Cochran Q statistic; *I^2^* = (Q-df)/Q×100%; *P* < 0.017 were considered statistically significant.

FEV1: forced expiratory volume in one second; FVC: forced vital capacity; FEV1/FVC: the ratio of FEV1 over FVC; AF: atrial fibrillation.

IVW: inverse-variance weighted; uvMR: univariable mendelian randomization; MR-PRESSO: Pleiotropy Residual Sum and Outlier; OR: odds ratio; SNP: single-nucleotide polymorphism.

Supplement Table 5. Pleiotropic test for the selected instrumental variable (AF)

| Outcome | Egger_intercept | SE | *P* value |
| --- | --- | --- | --- |
| FEV1 | 0.001 | 0.002 | 0.747 |
| FVC | -0.001 | 0.002 | 0.419 |
| FEV1/FVC | 0.002 | 0.002 | 0.199 |

FEV1: forced expiratory volume in one second; FVC: forced vital capacity; FEV1/FVC: the ratio of FEV1 over FVC; AF: atrial fibrillation; SE: standard error.

Supplement table 6. Multivariable MR analysis for lung function and AF

|  |  |  | | |  | | |  | | |  | | | Heterogeneity | | | MR-PRESSO | |
| --- | --- | --- | --- | --- | --- | --- | --- | --- | --- | --- | --- | --- | --- | --- | --- | --- | --- | --- |
| Exposure | nSNP | | | OR (95% CI) | | SE | | | *P* | | | Intercept *P* | | Q | Q_*p* | *I^2^* | *P* | RSS obs |
| FEV1 |  | |  | | | |  | | |  | | |  | 41.014 | 0.023 | 62.3% |  |  |
| mvIVW | 28 | | 0.501(0.056~4.457) | | | | 1.015 | | | 0.496 | | |  |  |  |  | 0.502 | 50.981 |
| mvMR-Egger | 28 | | 1.478(0.201~10.873) | | | |  | | |  | | | 0.886 |  |  |  |  |  |
| FVC |  | |  | | | |  | | |  | | |  |  |  |  |  |  |
| mvIVW | 28 | | 1.969(0.288~13.474) | | | | 0.981 | | | 0.490 | | |  |  |  |  | 0.496 |  |
| mvMR-Egger | 28 | | 1.247(0.335~4.512) | | | |  | | |  | | | 0.361 |  |  |  |  |  |
| FEV1/FVC |  | |  | | | |  | | |  | | |  |  |  |  |  |  |
| mvIVW | 28 | | 1.523(0.445~5.217) | | | | 0.628 | | | 0.503 | | |  |  |  |  | 0.509 |  |
| mvMR-Egger | 28 | | 0.800(0.091~7.033) | | | |  | | |  | | | 0.299 |  |  |  |  |  |

FEV1: forced expiratory volume in one second; FVC: forced vital capacity; FEV1/FVC: the ratio of FEV1 over FVC; AF: atrial fibrillation; SE: standard error. mvIVW: multivariable inverse-variance weighted; mvMR: multivariable mendelian randomization; MR-PRESSO: Pleiotropy Residual Sum and Outlier; OR: odds ratio; CI: confidence interval; SNP: single-nucleotide polymorphism; RSS obs: residual sums squares of observations.

# Supplementary Results of replication analysis

**Genetic IVs selection and validation (replication analyses)**

In the repetition univariable bidirectional MR analyses, we obtained 200/186/218/29 LD-independent (r^2^ < 0.0001) SNPs which achieved genome wide significance (*P* < 5×10^-8^) for FEV1, FVC, FEV1/FVC and AF, respectively, then we extracted those SNPs from corresponding outcome dataset. Finally, 167/161/186/25 SNPs remained after harmonizing the datasets of exposure and outcome (33/25/32/4 palindrome SNPs with intermediate allele frequencies were removed).

In mvMR analysis, all 604 (200+186+218) LD-independent SNPs associated with FEV1, FVC and FEV1/FVC were included. 51 of these SNPs represent the same signal, so we aggregate a list of SNP (553 = 604 - 51) combinations by selecting the SNPs with the lowest *P* value. Finally, 470 SNPs for lung function were obtained after pruning and harmonizing (83 SNPs were removed for being palindromic with intermediate allele frequencies).

The number of SNPs for FEV1 [variance explained (R^2^): 2.8%], FVC [variance explained (R^2^): 2.5%] and FEV1/FVC [variance explained (R^2^): 5.1%] and the F statistic estimate is larger than that of original results. The number of IVs and statistical power included for exposures were listed in Table R1, and details for the characteristics of SNPs used in MR analyses were shown in Supplementary material (Table 2.xlsx).

**Causal association of FEV1, FVC or FEV1/FVC with AF via forward MR (replication analyses)**

***1. FEV1***

All models suggested that FEV1 has no causal effect on AF (OR = 0.867, 95% CI = 0.742-1.014, *P* = 0.074) without evidence of pleiotropy effect (intercept = 0.004, *P* = 0.409) but moderate heterogeneity (Q_pval = 1.48e-06, *I^2^* = 37%). The leave-one-out sensitivity analysis and the MR-PRESSO test both detected the 2^nd^, 22^nd^, 33^rd^, 39^th^, 71^st^, 84^th^, 133^rd^ and 191^st^ SNP (rs116460699, rs4555304, rs2304340, rs3856802, rs11241689, rs12212123, rs7116641 and rs2206925) as pleiotropic outliers. The results of IVW show that higher FEV1 was associated with lower risk of AF (IVW: OR = 0.844, 95% CI = 0.735-0.969, *P* = 0.016; intercept = 0.003, *P* = 0.475), and heterogeneity was also reduced (Q_pval = 0.035, *I^2^* = 18%) after rerun the MR by removing the outlier SNPs (Table R2). As the *P* value is extremely close to the 0.17, mvMR analysis was performed and the results should be interpreted cautiously. The WM method estimates were more precise than the MR-Egger method (WM: OR = 0.867, 95% CI = 0.707-1.064, *P* = 0.173; MR Egger: OR = 0.725, 95% CI = 0.468-1.124, *P* = 0.509) (Table R2).

***2. FVC***

No evidence was detected for a causal relation between FVC and AF in IVW method (OR = 0.854, 95% CI = 0.725-1.006, *P* = 0.059) with no evidence of pleiotropy (intercept = -0.005, *P* = 0.372) but significant heterogeneity (Q_pval = 1.74e-05, *I^2^* = 35%). The leave-one-out sensitivity analysis and the MR-PRESSO test both detected the 5^th^, 28^th^, 47^th^, 104^th^, 121^st^ and 133^rd^ SNPs (rs2885697, rs72995681, rs17767210, rs2637254, rs1270884 and rs76219171) as pleiotropic outlier. Although significant heterogeneity was no longer exist (Q_pval = 0.029, *I^2^* = 18%) after rerun the MR by removing the outlier SNPs, the MR estimates did not alter significantly (IVW: OR = 0.906, 95% CI = 0.781-1.052, *P* = 0.197; intercept = -0.002, *P* = 0.659) (Table R2).

***3. FEV1/FVC***

All methods except WM suggested that FEV1/FVC has no causal effect on AF (IVW: OR = 1.024, 95% CI = 0.932-1.124, *P* = 0.622) with no evidence of pleiotropy (intercept = -0.002, *P* = 0.409) and heterogeneity (Q_pval = 0.471, *I^2^* = 0%). The leave-one-out sensitivity analysis and the MR-PRESSO test both detected the 195^th^ SNP as pleiotropic outlier. The MR estimates did not alter significantly (IVW: OR = 1.021, 95% CI = 0.930-1.122, *P* = 0.657; intercept = -0.003, *P* = 0.375) after rerun the MR by removing the outlier SNPs (Table R2).

**Causal association of AF with FEV1, FVC and FEV1/FVC via reverse MR (replication analyses)**

All models in reverse MR analyses consistently suggested that genetically instrumented AF has no significant correlation with FEV1 (OR = 0.991, 95% CI = 0.980-1.002, *P* = 0.124), FVC (OR = 0.947, 95% CI = 0.878-1.022, *P* = 0.161) and FEV1/FVC (OR = 0.997, 95% CI = 0.896-1.110, *P* = 0.956). Pleiotropy bias (intercept_FEV1_ = 0.001, *P*_FEV1_ = 0.656; intercept_FVC_ = 0.001, *P*_FVC_ = 0.664; intercept_FEV1/FVC_ = 0.006, *P*_FEV1/FVC_ = 0.190) was not detected, and heterogeneity (FEV1: Q_pval = 0.014, *I^2^* = 43%; FVC: Q_pval = 0.602, *I^2^* = 0%; FEV1/FVC: Q_pval = 0.282, *I^2^* = 17%) was lower by removing the potential outliers (FEV1: rs147790633; FVC: rs539526, rs6957206, rs147790633; FEV1/FVC:rs880315/rs9825233/rs7633500/rs529526/rs3807989/rs6957206/rs147790633/rs2209073/rs12600452/rs8108692) based on the leave-one-out and MR-PRESSO analyses. These main estimates from IVW were broadly consistent with estimates from the WM and MR-Egger sensitivity analyses. All reverse leave-one-out sensitivity analyses and the MR-PRESSO tests after removing the outliers indicated that the correlation between AF with FEV1, FVC or FEV1/FVC was not significantly affected by any individual SNP, indicating the robustness of the reverse MR analyses results (Table R3).

**Causal association of lung function with AF via mvMR approach (replication analyses)**

The estimate effects from the mvMR analysis showed that the direct effect of FVC or FEV1/FVC (OR_FVC_ = 2.983, 95% CI = 0.249-35.602, *P* = 0.387; OR_FEV1/FVC_ = 0.375, 95% CI = 0.038-3.693, *P* = 0.400) controlling for another two lung function arguments was similar with the univariable setting. Additionally, no effect of FEV1 (OR = 0.608, 95% CI = 0.866-1.978, *P* = 0.409) on AF could be observed, which was inconsistent with the univariable MR IVW result. Consistent with standard IVW regression results, mvMR-Egger regression (orientated to FEV1/FVC) and MR-PRESSO (detect no significant outlier) results also showed no significant association between lung function and AF, suggesting that the mvMR method successfully overcomes the bias caused by horizontal pleiotropy in the univariable MR analysis (Table R4).

# Supplementary Tables of replication results

Table R1. Baseline information of exposures used in our analyses

| Exposure |  | Outcome |  | Number of IVs | | R^2^ | | Power (%) | | F_statistics | |
| --- | --- | --- | --- | --- | --- | --- | --- | --- | --- | --- | --- |
| Trait | Sample | Trait | Sample | uvMR | mvMR | uvMR | mvMR | uvMR | mvMR | uvMR | mvMR |
| FEV1 | 400,462 | AF | 218,792 | 167 | 470 | 0.028 | | 91 | | 11396.56 | |
| FVC | 400,462 | AF | 218,792 | 161 |  | 0.025 | 0.096 | 39 | 100 | 10068.63 | 38551.63 |
| FEV1/FVC | 400,462 | AF | 218,792 | 186 |  | 0.051 | | 4 | | 20272.48 | |
| AF | 218,792 | FEV1 | 400,462 | 24 | - | 0.175 | | 100 | | 38817.22 | |
| AF | 218,792 | FVC | 400,462 | 19 | - | 0.149 | | 100 | | 33527.58 | |
| AF | 218,792 | FEV1/FVC | 400,462 | 12 | - | 0.117 | | 100 | | 26493.10 | |

FEV1: forced expiratory volume in one second; FVC: forced vital capacity; FEV1/FVC: the ratio of FEV1 over FVC; AF: atrial fibrillation; IVs: instrumental variables; R^2^: Variance explained by the SNPs on exposure.

Table R2. Forward causal relations of FEV1, FVC and FEV1/FVC with AF performed by uvMR

| Exposure | nSNPs | OR (95%CI) | *P* | Q_pval(*I^2^*) | Intercept(*P*) | Global *P* |
| --- | --- | --- | --- | --- | --- | --- |
| FEV1 vs. AF |  |  |  |  |  |  |
| IVW | 159 | 0.844(0.735-0.969) | 0.016 | 0.035(0.18) |  |  |
| MR-Egger | 159 | 0.725(0.468-1.124) | 0.153 |  | 0.003(0.475) |  |
| WM | 159 | 0.867(0.705-1.067) | 0.178 |  |  |  |
| MR-PRESSO | 159 |  | 0.085 |  |  | 0.016 |
| FVC vs. AF |  |  |  |  |  |  |
| IVW | 155 | 0.906(0.781-1.052) | 0.197 | 0.029(0.18) |  |  |
| MR-Egger | 155 | 1.014(0.604-1.701) | 0.959 |  | -0.002(0.659) |  |
| WM | 155 | 0.896(0.734-1.093) | 0.279 |  |  |  |
| MR-PRESSO | 155 |  | 0.073 |  |  | 0.002 |
| FEV1/FVC vs. AF |  |  |  |  |  |  |
| IVW | 185 | 1.021(0.930-1.122) | 0.657 | 0.469(0.00) |  |  |
| MR-Egger | 185 | 1.111(0.903-1.368) | 0.322 |  | -0.003(0.375) |  |
| WM | 185 | 1.232(1.053-1.441) | 0.009 |  |  |  |
| MR-PRESSO | 185 |  | 0.592 |  |  | <2e-04 |

nSNPs: number of single nucleotide polymorphisms; OR: odds ratio; CI: confidence interval; Q_pval: P value of the Cochran Q statistic; *I^2^* = (Q-df)/Q×100%; *P* < 0.017 were considered statistically significant. FEV1: forced expiratory volume in one second; FVC: forced vital capacity; FEV1/FVC: the ratio of FEV1 over FVC; AF: atrial fibrillation. uvMR: univariable mendelian randomization; IVW: inverse-variance weighted; WM: weighted median; MR-PRESSO: Pleiotropy Residual Sum and Outlier.

Table R3. Reverse causal relations of AF with FEV1, FVC and FEV1/FVC performed by uvMR

| Exposure | nSNPs | OR (95%CI) | *P* | Q_pval(*I^2^*) | Intercept(*P*) | Global *P* |
| --- | --- | --- | --- | --- | --- | --- |
| AF vs. FEV1 |  |  |  |  |  |  |
| IVW | 24 | 0.991(0.980-1.002) | 0.124 | 0.014(0.43) |  |  |
| MR-Egger | 24 | 0.986(0.962-1.011) | 0.290 |  | 0.001(0.656) |  |
| WM | 24 | 0.987(0.975-0.999) | 0.048 |  |  |  |
| MR-PRESSO | 24 |  | 0.229 |  |  | 0.013 |
| AF vs. FVC |  |  |  |  |  |  |
| IVW | 19 | 0.947(0.878-1.022) | 0.161 | 0.602(0.00) |  |  |
| MR-Egger | 19 | 0.872(0.599-1.269) | 0.483 |  | 0.001(0.664) |  |
| WM | 19 | 0.946(0.847-1.057) | 0.312 |  |  |  |
| MR-PRESSO | 19 |  | 0.416 |  |  | 0.541 |
| AF vs. FEV1/FVC |  |  |  |  |  |  |
| IVW | 12 | 0.997(0.896-1.110) | 0.956 | 0.282(0.17) |  |  |
| MR-Egger | 12 | 0.732(0.470-1.139) | 0.197 |  | 0.006(0.190) |  |
| WM | 12 | 0.982(0.822-1.172) | 0.837 |  |  |  |
| MR-PRESSO | 12 |  | 0.170 |  |  | 0.855 |

nSNPs: number of single-nucleotide polymorphisms; OR: odds ratio; CI: confidence interval; Q_pval: *P* value of the Cochran Q statistic; *I^2^* = (Q-df)/Q×100%; *P* < 0.017 were considered statistically significant. FEV1: forced expiratory volume in one second; FVC: forced vital capacity; FEV1/FVC: the ratio of FEV1 over FVC; AF: atrial fibrillation. uvMR: univariable mendelian randomization; IVW: inverse-variance weighted; WM: weighted median; MR-PRESSO: Pleiotropy Residual Sum and Outlier.

Table R4. Multivariable MR analyses for lung function and AF

| Exposure | nSNPs | OR (95% CI) | *P* | Intercept *P* | | Q_pval (*I^2^*) | PRESSO *P* |
| --- | --- | --- | --- | --- | --- | --- | --- |
| FEV1 |  |  |  |  | | 0.473(0.002) |  |
| mvIVW | 470 | 0.608(01886~1.978) | 0.409 |  | |  | 0.274 |
| mvMR-Egger | 470 | 2.959(0.247~35.388) |  |  | |  |  |
| FVC |  |  |  | |  |  |  |
| mvIVW | 470 | 2.983(0.249~35.602) | 0.387 | 0.000 | |  | 0.245 |
| mvMR-Egger | 470 | 0.378(0.038~3.726) |  |  | |  |  |
| FEV1/FVC |  |  |  |  | |  |  |
| mvIVW | 470 | 0.375(0.038~3.693) | 0.400 |  | |  | 0.269 |
| mvMR-Egger | 470 | 0.616 (0.189~2.013) |  |  | |  |  |

FEV1: forced expiratory volume in one second; FVC: forced vital capacity; FEV1/FVC: the ratio of FEV1 over FVC; AF: atrial fibrillation; SE: standard error. mvIVW: multivariable inverse-variance weighted; mvMR: multivariable mendelian randomization; MR-PRESSO: Pleiotropy Residual Sum and Outlier; OR: odds ratio; CI: confidence interval; nSNPs: number of single nucleotide polymorphisms.
